# Heparan Sulfate Proteoglycans as Drivers of Neural Progenitors Derived From Human Mesenchymal Stem Cells

**DOI:** 10.3389/fnmol.2018.00134

**Published:** 2018-04-24

**Authors:** Rachel K. Okolicsanyi, Lotta E. Oikari, Chieh Yu, Lyn R. Griffiths, Larisa M. Haupt

**Affiliations:** Genomics Research Centre, Institute of Health and Biomedical Innovation, School of Biomedical Sciences, Queensland University of Technology, Brisbane, QLD, Australia

**Keywords:** heparan sulfate proteoglycans, mesenchymal stem cell induced neurospheres, neural potential, glypican, syndecan, neural stem cell

## Abstract

**Background:** Due to their relative ease of isolation and their high *ex vivo* and *in vitro* expansive potential, human mesenchymal stem cells (hMSCs) are an attractive candidate for therapeutic applications in the treatment of brain injury and neurological diseases. Heparan sulfate proteoglycans (HSPGs) are a family of ubiquitous proteins involved in a number of vital cellular processes including proliferation and stem cell lineage differentiation.

**Methods:** Following the determination that hMSCs maintain neural potential throughout extended *in vitro* expansion, we examined the role of HSPGs in mediating the neural potential of hMSCs. hMSCs cultured in basal conditions (undifferentiated monolayer cultures) were found to co-express neural markers and HSPGs throughout expansion with modulation of the *in vitro* niche through the addition of exogenous HS influencing cellular HSPG and neural marker expression.

**Results:** Conversion of hMSCs into hMSC Induced Neurospheres (hMSC IN) identified distinctly localized HSPG staining within the spheres along with altered gene expression of HSPG core protein and biosynthetic enzymes when compared to undifferentiated hMSCs.

**Conclusion:** Comparison of markers of pluripotency, neural self-renewal and neural lineage specification between hMSC IN, hMSC and human neural stem cell (hNSC H9) cultures suggest that *in vitro* generated hMSC IN may represent an intermediary neurogenic cell type, similar to a common neural progenitor cell. In addition, this data demonstrates HSPGs and their biosynthesis machinery, are associated with hMSC IN formation. The identification of specific HSPGs driving hMSC lineage-specification will likely provide new markers to allow better use of hMSCs in therapeutic applications and improve our understanding of human neurogenesis.

**GRAPHICAL ABSTRACT:**
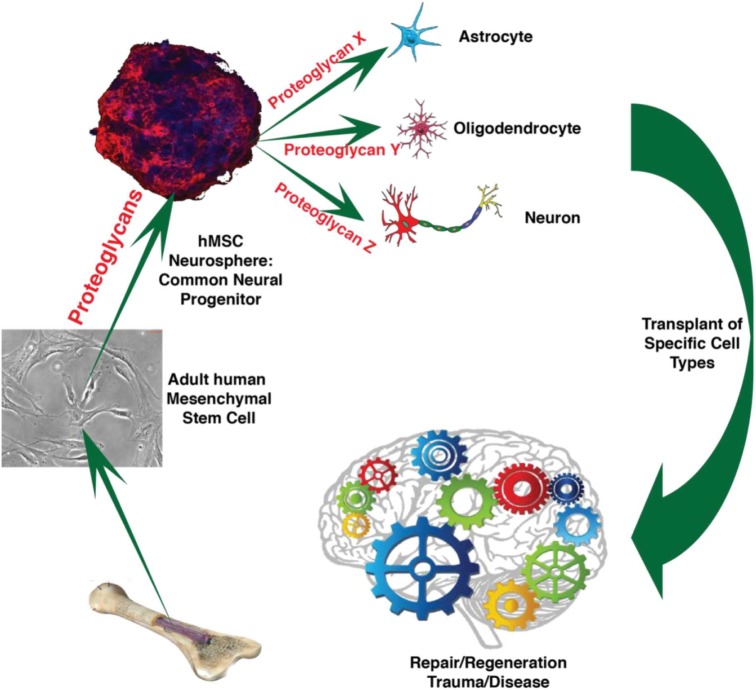
Human mesenchymal stem cells (hMSC) produce common neural progenitor-like hMSC induced neurospheres (hMSC IN). We postulate that proteoglycans, specifically heparan sulfate proteoglycans, are instrumental to convert hMSC to hMSC IN and that hMSC IN are equivalent to common neural progenitor cells retaining neural differentiation capacity to produce critical neural cell types for therapy following neurodegeneration triggered by trauma or disease. (Image parts from: Ivins et al., 1997; Johnson et al., 2007; Eatman, 2011; Williams, 2016).

## Introduction

Brain damage, whether acquired through injury or disease, affects people of all ages with subsequent neuronal degeneration variable and structural damage evident even when the initial injury appears to be mild (Jenkins et al., 1986; Fortune and Wen, 1999; Choe, 2016; Stocchetti and Zanier, 2016; Ganos et al., 2017). Human mesenchymal stem cells (hMSCs) traditionally isolated from the bone marrow can also be isolated from a number of other sources including fat, umbilical cord blood and dental pulp (Mafi et al., 2011; Okolicsanyi et al., 2014, 2015). The multipotential nature of hMSCs along with their relative ease of isolation and high *ex vivo* expansive potential continues to make these cells attractive for therapeutic applications, including the treatment of brain trauma and neurodegenerative diseases (Minguell et al., 2001; Hermann et al., 2006; Ragni et al., 2013; Abdullah et al., 2016).

Proteoglycans (PGs) consist of a core protein to which unbranched high molecular weight glycosaminoglycan (GAG) side chains attach (Yu et al., 1995; Bandtlow and Zimmermann, 2000; Hacker et al., 2005; Ori et al., 2008; Sarrazin et al., 2011; Okolicsanyi et al., 2014; Oikari et al., 2016b). The heparan sulfate family of proteoglycans (HSPGs) includes four transmembrane syndecans (SDC1-4), and six GPI-anchored glypicans (GPC1-6) (Bernfield et al., 1999; Tkachenko et al., 2005; Leonova and Galzitskaya, 2013). These diverse proteins are major constituents of the extracellular matrix (ECM), the cell and its microenvironment (intracellular compartments, cell surface and basement membranes) that structurally and functionally influence the cellular functions of proliferation, differentiation and gene expression (Bandtlow and Zimmermann, 2000; Sarrazin et al., 2011). With the linked GAG chains responsible for much of the biological role of HSPGs, the core protein functions to maximize GAG chain efficiency through diverse regulatory interactions (Bernfield et al., 1999; Matsuo and Kimura-Yoshida, 2014) with growth factors and morphogens (FGF, Wnts, BMPs), their receptors (FGFRs), and ECM structural molecules (collagen, fibronectin) (Bernfield et al., 1999; Habuchi et al., 2004; Hacker et al., 2005; Sarrazin et al., 2011). As such, through localization and function, HSPGs are central modulators of protein gradient formation and signal transduction within the localized cellular microenvironment.

Neural development is a complex process comprised of successive phases of cell migration and differentiation associated with regulatory signaling events (Wilson and Edlund, 2001; Choi et al., 2006). SDCs influence cell adhesion, proliferation and differentiation (Hanahan and Weinberg, 2000; Wolf and Friedl, 2006; Okolicsanyi et al., 2014), with GPC proteins widely expressed throughout the central nervous system (CNS) during development demonstrated to stimulate and inhibit signaling activity (David, 1993; Lander et al., 1996; Fransson, 2003). HSPG activity identified during neural development (Yamaguchi, 2001) and neural lineage specification (Pickford et al., 2011) appears to be dependent on temporal developmental expression along with interactions with appropriate signaling pathways through core proteins and HS chain-specific sulfation (Yamaguchi, 2001).

Heparan sulfate dependent interactions of the BMP, Wnt and FGF signaling pathways have been suggested to regulate neural specification (Bally-Cuif and Hammerschmidt, 2003; Choi et al., 2006). In particular, the HS ligand FGF2 has a central role during proliferation and differentiation of murine neural stem cells in the developing cerebral cortex (Powell et al., 1991; Giordano et al., 1992; Yamaguchi, 2001; Wiese et al., 2004), confirming the importance of HSPGs in defining neural cell progenies (Qian et al., 1997; Yamaguchi, 2001).

Together with their extensive capacity for self-renewal, highly malleable mesenchymal stem cells (MSCs) give rise to diverse differentiated progenies, including neural lineages, and have important regenerative therapeutic potential for numerous applications including the treatment of brain trauma and neurological disorders (Galindo et al., 2011). Although expression of neural markers in MSCs in their undifferentiated state is now well documented in human and murine models (Bossolasco et al., 2005; Hermann et al., 2006; Alexanian, 2010; Fricke et al., 2014; Okolicsanyi et al., 2014, 2015; Li et al., 2015; Marinowic et al., 2015), in order to fully exploit their neurological regenerative potential, the identification of key genes regulating these processes is needed. We have previously examined commercially available hMSC donor populations for their expansive potential and identified key growth phases along with the maintenance of stemness and multipotentiality during extended expansion *in vitro* (Okolicsanyi et al., 2015). Here we examined the potential role of HSPGs in mediating hMSC neural specification during hMSC induced neurosphere formation, a preliminary stage of hMSC neural differentiation.

## Materials and Methods

### Cell Culture

Human mesenchymal stem cell (hMSC, *n* = 3) populations (Okolicsanyi et al., 2015) and human neural stem cells (hNSC H9) (Oikari et al., 2016b) were cultured under basal conditions as previously described. These cells were obtained through informed consent (see manufacturer’s supporting documentation (USWV-10276) with no additional ethical approval needed for this study and have been used previously (Okolicsanyi et al., 2015).

### *In Vitro* Niche Modification

Heparin is a short, highly sulfated protein analog of heparan sulfate (HS) with a several-fold higher degree of polymerization and more extensive modification than HS (Sugahara and Kitagawa, 2002) routinely used in cell culture and *in vitro* models as a HS substitute. Sodium chlorate competitively inhibits the formation of the high-energy sulfate donor in cellular sulfation reactions (Rapraeger et al., 1991), inhibiting further sulfation of the GAG chains. Treatment of cultures with 50 mM chlorate has been shown to inhibit overall *O*-sulfation of HS by ∼ 70% with *N*-sulfation remaining unchanged (Safaiyan et al., 1999), with lower concentrations of sodium chlorate (5–20 mM) shown to selectively reduce the 6-*O*-sulfation and a concentration of 50 mM to reduce 2-*O-* and 6-*O-*sulfation (Safaiyan et al., 1999). Modulation of the *in vitro* niche was performed by the addition of exogenous heparin or sodium chlorate to cells plated in 24-well plates (Corning, Australia) in varying concentrations: heparin (Sigma Aldrich, Australia) (0, 1, 5, 10, 25, and 50 μg/mL); sodium chlorate (Sigma Aldrich) (0, 1, 5, 50, 100, and 500 mM). In the dose response experiment, cells were monitored for 5 days with samples collected at day 1 (D1), day 3 (D3) and day 5 (D5) for RNA and protein isolation along with quantitation of cell number and viability (**Supplementary Figure S1**). In all subsequent experiments a concentration of 10 μg/mL heparin or 50 mM sodium chlorate was used, with cells grown in triplicate (*n* = 9) and maintained for 3 days followed by RNA extraction and Q-PCR analysis.

### hMSC Induced Neurosphere (hMSC IN) Formation

hMSC IN were developed in basal hMSC cultures at growth phase A (P+5) as described previously (Okolicsanyi et al., 2015). Briefly, 3–5 × 10^6^ hMSCs were plated to low attachment culture dishes following dissociation of the monolayer. Cells were plated in knockout DMEM/F12 supplemented with 20 ng/mL each of EGF and FGF and 10 μg/mL heparin. hMSC IN were observed after a few hours. Media and growth factors were replaced after 3 days following removal and filtering (40 μm filter; Greiner Bio-One, Germany) to retain cells. Growth factors were further supplemented at D5 and hMSC IN harvested for subsequent analysis after 7 days.

### RNA Isolation, Reverse Transcription and Q-PCR

RNA was isolated from hMSC and hNSC cultures as previously described (Okolicsanyi et al., 2015; Oikari et al., 2016b). Specific primer sequences for all genes investigated are summarized in **Supplementary Table S1**. Q-PCR cycling and reaction conditions have been described previously (Okolicsanyi et al., 2015; Oikari et al., 2016b). All Q-PCR experiments on undifferentiated cells were performed in quadruplicate on biological triplicates. Q-PCR on hMSC IN and hNSC H9 were conducted in quadruplicate. Variation between hMSCs, hMSC IN and hNSC H9 were assessed using a two-tailed unpaired Student’s *T*-test assuming unequal variance. Statistical significance was set to α = 0.05. Mean gene expression (2^-ΔΔCt^) is presented with standard error of the mean (SEM). Differences in hMSC gene expression between dose (heparin) and control were also assessed using a two-tailed unpaired Student’s *T*-test. Significance was set at α = 0.05. All gene Q-PCR expression was normalized to the endogenous control 18*S*, determined as the appropriated endogenous control for hMSC cultures as previously described (Haupt et al., 2009; Okolicsanyi et al., 2015; Oikari et al., 2016a,b).

### Immunocytochemistry of hMSCs and Confocal Microscopy of hMSC IN

Immunocytochemistry of hMSC cultures was performed as previously described (Okolicsanyi et al., 2015). For confocal microscopy, hMSC IN were plated in 8-chamber slides (Nunc Lab-Tek II CC2) and allowed to attach for 20 min. Half of the media was then carefully replaced with 4% PFA (final 2% PFA) and hMSC IN fixed for 20 min before the medium/PFA mix was replaced with 500 μL 4% PFA. hMSC IN were fixed for a further 30 min at RT (static), before being carefully washed with 1X PBS and stored at 4°C in 1X PBS until staining. Blocking was conducted in 5% NDS, 0.3% Triton X-100 in 1X PBS for 1 h. Primary antibodies were diluted in blocking solution and cultures incubated overnight at 4°C followed by three 5 min washes in blocking solution containing 0.03% Triton-X-100 and the slides re-blocked for 30 min followed by 1-h static incubation at RT with secondary antibody solution. Secondary antibodies and concentrations have been described previously (Okolicsanyi et al., 2015). Slides then underwent post-fixation (4% PFA, 20 min) and glycine-quenching (100 mM Glycine/PBS, 20 min) prior to mounting with Fluoroshield anti-fade mounting medium containing DAPI (Abcam). hMSC IN were imaged using a Leica SP5 confocal system with Z-stacks created from slices taken at 0.8 μm intervals and maximum intensity projections generated at post-processing. 3D reconstructions were created using Volocity v6.3 (Perkin-Elmer). All washing and blocking steps were conducted with gentle rocking unless otherwise stated.

## Results

### HSPG Gene Expression Correlates With Markers of Self-Renewal and Neural Lineages

Gene expression of SDC and GPC core proteins were examined under basal culture conditions at previously identified growth phases (A-C) with the plateau growth phase (Phase D) (Okolicsanyi et al., 2015) not included in this study. Gene expression of SDC1-3 increased throughout expansion (Phase A-C) with these SDC genes displaying a relative twofold increase in gene expression by Phase C (**Supplementary Figure S2A**). In contrast, gene expression of SDC4 remained low throughout expansion with levels detected at Phase C at approximately 50% of the levels of SDC1 observed at Phase A (**Supplementary Figure S2A**).

GPC1 demonstrated a twofold increase in expression by Phase C when compared to Phase A cells (**Supplementary Figure S2B**). Expression of GPC2 and GPC3 was detected at intermediate (Phase B) and late (Phase C) phases of growth, however, expression levels of these genes were observed to be less than 10% of GPC1 levels at corresponding growth phases (**Supplementary Figure S2B**). GPC2 and GPC3 demonstrated a 1–2 fold increase in gene expression by growth Phase C. GPC4 and GPC6 exhibited low levels of gene expression throughout expansion, with a moderate increase observed in Phase B cultures. The gene expression level of GPC4 was observed to be 60% lower than the level of GPC1 in the early phase of growth (Phase A) with GPC5 not expressed.

**FIGURE 1:**
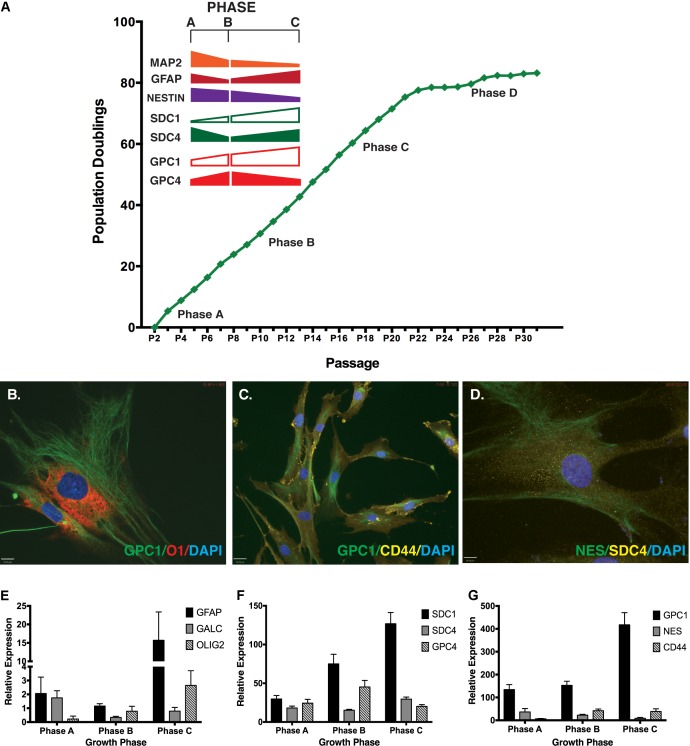
Correlation of neural markers with heparan sulfate proteoglycan (HSPG) core proteins throughout extended human mesenchymal stem cell (hMSC) *in vitro* expansion. **(A)** hMSC populations were expanded for >80 population doublings *in vitro* and gene expression of key HSPG core proteins correlated with key neural markers throughout different phases of growth (Phase A-C). **(B–D)** Representative immunocytochemistry (ICC) images of neural stemness and lineage markers simultaneously expressed with key HSPG core proteins in undifferentiated hMSCs. Primary antibodies against each protein were used. Secondary antibodies: FITC: green; Cy3: yellow; and AF594: red. All cultures were counterstained with DAPI to identify nuclei. Scale bars represent 70 μm. **(E–G)** Q-PCR gene expression of **(E)** specific glial lineage markers: GFAP, GALC and OLIG2 **(F)** HSPG core proteins: SDC1, SDC4, and GPC4 **(G)** HSPG core protein GPC1, with neural stemness (Nestin) and glial (CD44) markers.

Neural stem cell self-renewal and neural lineage markers examined in basal culture conditions included: Nestin and SOX2 (self-renewal), MAP2 (neuronal lineage), GFAP (astrocyte lineage) and GalC (oligodendrocyte lineage). Throughout expansion several neural markers displayed similar gene expression patterns to those observed for the HSPG core proteins (**Figure 1A**). Strong positive staining was observed for markers co-localized in undifferentiated cultures by ICC (**Figures 1B–D**) including: GPC1 and O1 (oligodendrocyte; **Figure 1B**); GPC1 and CD44 (MSC marker; **Figure 1C**), Nestin and SDC4 (**Figure 1D**). Gene expression was validated by Q-PCR (**Figures 1E–G**). Similarities between gene expression patterns were seen between SDC4, and glial markers GFAP and GalC with all three proteins demonstrating the lowest expression level in Phase B cultures with less than 50% of the expression than observed at Phase C (**Figures 1E–G**). SDC1, GPC1 and Olig2 (glial, oligodendrocyte) were all observed to increase gene expression throughout expansion with GPC4 and CD44 demonstrating the highest level of gene expression at Phase B (**Figures 1E–G**). Glial lineage markers GalC, Olig2, GFAP (**Figure 1E**) and the neuronal marker, TUBB3 (Okolicsanyi et al., 2015) were detected at each phase of growth (Phases A–C). Neural self-renewal markers (Nestin) and the astrocyte lineage [GFAP, CD44; (Liu et al., 2004; Naruse et al., 2013; Sosunov et al., 2014; Oikari et al., 2016a)] were expressed at similar levels to SDC4 across all growth phases. In contrast, while expression of GalC and Olig2 (oligodendrocyte; **Figure 1E**) were observed throughout expansion, the level of Olig2 gene expression was less than 10% of the level observed for SDC4, indicative of terminally differentiated glial lineages.

Previous ICC staining of self-renewal and neural lineage markers identified homogeneous staining of SOX2 and GFAP in hMSC cultures. Nestin and the late neuronal marker MAP2 displayed reduced heterogeneous localization (<2% positive MAP2 staining (Okolicsanyi et al., 2015)) at all growth phases.

### Proliferative and Gene Expression Response to Niche Modification

**FIGURE 2:**
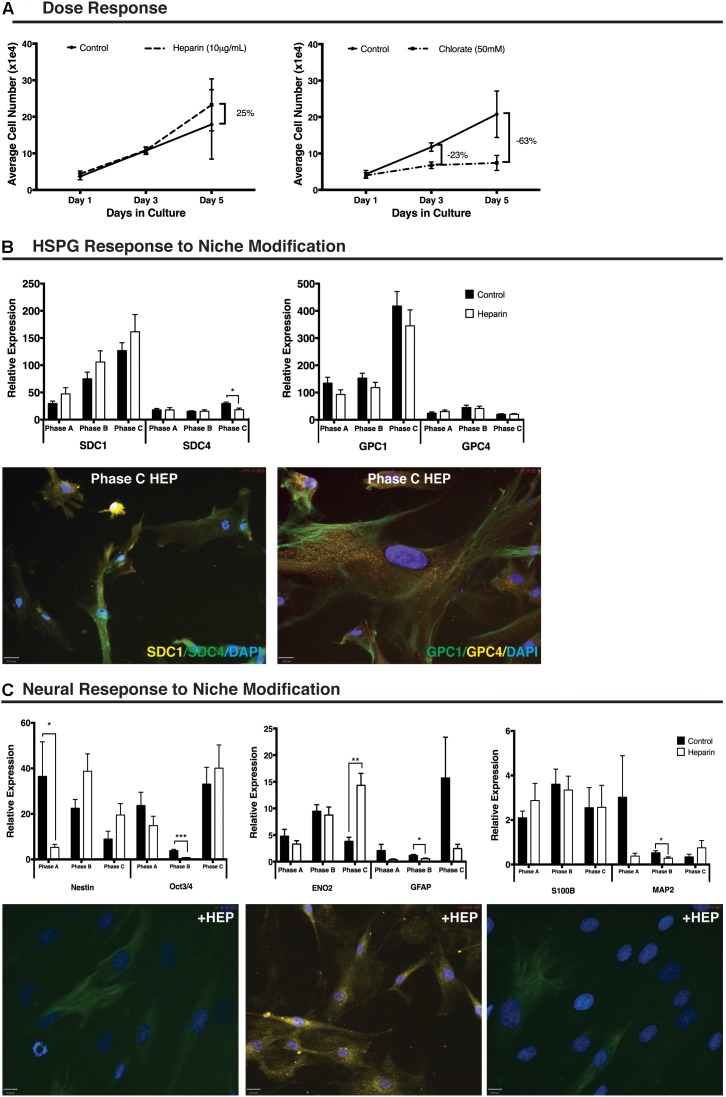
Human mesenchymal stem cell (hMSC) and heparan sulfate proteoglycan (HSPG) response to niche modification. **(A)** Dose response curve showing proliferation of undifferentiated hMSCs following addition of exogenous heparin (10 μg/mL) resulting in a 25% increase in cell number over control between day 3 and day 5. Treatment with exogenous sodium chlorate (50 mM) resulted in a 23% decrease in proliferation below control by day 3 and a 63% decrease in cell number below control by day 5. Control culture, solid black line; heparin treated culture, dashed line, sodium chlorate treated culture, dash-dot line. **(B)** Heparan sulfate proteoglycan (HSPG) core protein (SDC1, SDC4, GPC1, GPC4) response to niche modification at each growth phase. Following niche modification (10 μg/mL heparin) a significant decrease in SDC4 expression was observed at Phase C. All other changes were non-significant. Immunocytochemistry (ICC) staining revealed no obvious visual differences between control and heparin treated cultures matching corresponding Q-PCR data. Representative ICC images from heparin treated cultures at growth Phase C. **(C)** Neural response to niche modification. Following heparin treatment, pluripotency and stemness markers demonstrate significantly decreased expression (Nestin, Phase A; Oct3/4, Phase B) at early growth phases and increased expression at Phase C with the increase for ENO2 moderately significant. Glial markers (GFAP, S100B) demonstrated no significant differences in expression following heparin treatment except at Phase B where a significant decrease in GFAP expression was observed. The late neuronal marker MAP2 and the glial marker S100B were detected at levels 10–50% of other neural markers examined with a significant decrease in gene expression observed for MAP2 at growth Phase B. Primary antibodies detected individual proteins with secondary antibodies FITC (green) and Cy3 (yellow). Nuclei were counterstained with DAPI. Scale bars represent 70 μm. ^∗^*p* < 0.05, ^∗∗^*p* < 0.005, ^∗∗∗^*p* < 0.0001.

Treatment of cultures with 10 μg/mL of heparin increased cell number (25%) when compared with control cultures between day 3 (D3) and day 5 (D5) of treatment (*n* = 9). Treatment of cultures with 50 mM sodium chlorate resulted in a reduction in cell number (23%) when compared to control cultures between day 1 (D1) and D3. By D5 the overall cell number was reduced by 63% in the chlorate treated cultures when compared to control cultures (**Figure 2A**). For this study, further *in vitro* niche modification was limited to the addition of exogenous heparin to cultures due to the extreme reduction in cellular proliferation observed in the presence of sodium chlorate.

A non-significant increase in SDC1 gene expression was demonstrated following heparin treatment at each growth phase examined. Expression levels of SDC4 remained unchanged in early and intermediate-phases of growth (Phases A and B) following heparin treatment with a significant decreased gene expression observed in Phase C cells (*p* = 0.006). SDC4 gene expression remained at <50% of the levels observed for SDC1 in control cultures (**Figure 2B**). GPC1 demonstrated a non-significant decreased gene expression at all growth phases following addition of heparin (**Figure 2B**) with no significant changes observed in GPC4 gene expression at any growth phase with levels of this gene maintained at approximately 10% of GPC1 (**Figure 2B**).

ICC staining supported Q-PCR gene expression results with clear positive staining at all growth phases under basal and proliferative (heparin) culture conditions. Staining revealed heterogeneous localization of SDC1, with varying levels of signal intensity observed (**Figure 2B**). Under all culture conditions, strong staining of all cells was observed for both GPC1 and GPC4 (**Figure 2B**) with GPC1 demonstrating distinct localization and a filamentous expression pattern while GPC4 produced more homogenous and diffuse localization.

In response to heparin treatment, Nestin (self-renewal) demonstrated significantly reduced gene expression at growth phase A (*p* = 0.047) and non-significant increases in expression at growth phase B and C. The astrocyte lineage marker, GFAP (*p* = 0.006; **Figure 2C**) and the neuronal lineage marker, MAP2 (*p* = 0.04; **Figure 2C**), also demonstrated significantly reduced expression following heparin treatment at growth phase B. ICC examination of these cultures highlighted the heterogeneity of the expression of these markers within the cultures with GFAP staining observed in >90% of cells within the cultures. In contrast <50% of the cultures stained positive for Nestin, and <20% for MAP2 (**Figure 2C**) under control (Okolicsanyi et al., 2015) and proliferative conditions.

Q-PCR of neural lineage markers revealed reduced gene expression of the pluripotency marker OCT3/4 following addition of heparin at early (non-significant) and intermediate growth phases (Phase B: *p* = 5.4 × 10^-7^; **Figure 2C**) with a non-significant increase observed at Phase C. This pattern was duplicated for the neuronal marker enolase 2 (ENO2), with increased expression at Phase C highly significant (*p* = 0.0003; **Figure 2C**). The neuronal marker MAP2, demonstrated reduced expression following addition of heparin at Phase A (non-significant) and B (*p* = 0.04) and a non-significant increase at Phase C (**Figure 2C**) with no significant changes in gene expression of the astrocyte marker S100B. Expression of the oligodendrocyte lineage markers, GalC and Olig2, demonstrated non-significant reductions in expression at Phase A, and non-significant increases in expression at Phase B. Olig2 expression was further increased following addition of heparin to the cultures, however, these changes were not significant (**Supplementary Figure S3A**). Examination of additional neuronal lineage markers revealed a significantly reduced level of gene expression of Neural Cadherin (NCAD) at Phase C (*p* = 4.1 × 10^-7^) and TUBB3 in Phase A (*p* = 3.7 × 10^-5^) and Phase B (*p* = 0.002) cultures (**Supplementary Figure S3B**).

### hMSC Induced Neurosphere (hMSC IN) Formation

**FIGURE 3:**
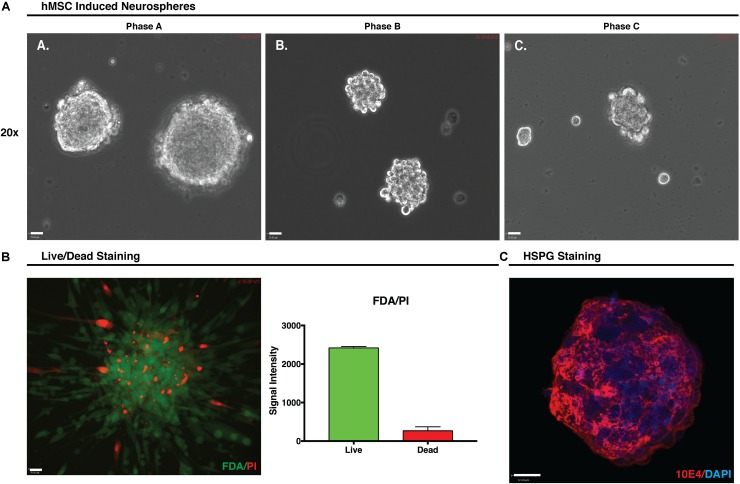
Human mesenchymal stem cell (hMSC) induced neurospheres. **(A)** Formation of hMSC-induced neurospheres (hMSC IN) at each growth phase. Spheres generated at Phase A were larger and more uniform in shape than those generated at Phase B or C. Small, uneven cell clusters rather than spheres were observed at Phase C. Scale bars represent 70 μm. **(B)** Phase A hMSC IN were examined using FDA/PI stain to determine the live/dead cell ratio. Relative signal intensity demonstrated hMSC IN were predominantly composed of live cells with confocal imaging confirming live cells were distributed throughout the sphere and were not restricted to the sphere surface. Scale bar represents 70 μm. **(C)** Phase A spheres stained using pan-heparan sulfate (10E4) primary antibody detecting HS chains, irrespective of the core protein to which they are attached. Secondary antibody used was AF594. Nuclei were counterstained with DAPI. Staining clearly reveals high levels of both heparan sulfate in Phase A spheres. Scale bar represents 12 μm.

During hMSC IN formation, at all growth phases, clustering of cells was observed within an hour and sphere formation became apparent after only a few hours in induction media. By 24 h the presence of large numbers of hMSC IN of varying sizes was observed in Phase A and B cultures where large healthy hMSC IN formed in all populations. In Phase C cultures, cells predominantly clustered rather than formed spheres with smaller (>50%) and less uniform in shape hMSC IN observed (**Figure 3A**). The diameter of the hMSC IN varied with the maximum diameters > 400 μm observed in Phase A cultures; a maximum diameter of approximately 300 μm in Phase B cultures; and a maximum sphere diameter of approximately 200 μm with the majority of cell clusters observed to be no more than 100 μm in diameter in Phase C cultures. Fewer hMSC IN were formed in Phase B cultures when compared to Phase A cultures (∼70–80%). In addition to their reduced ability to form hMSC IN and the smaller sphere diameter observed, media changes at D3 post-induction resulted in the Phase C hMSC IN disintegrating into smaller cell clusters. This process had no effect on the integrity of the hMSC IN generated in Phase A and B cultures.

To examine the effect of inhibition of further sulfation of the GAG chains, heparin in the induction media was replaced with 50 mM sodium chlorate, resulting in small and irregular shaped spheres when compared to those formed in the presence of heparin. These spheres were not deemed sufficiently stable and viable and as such were deemed unsuitable for use in further *in vitro* modification experiments. With hMSC IN formed in hMSC Phase A-C cultures, the remainder of this study focusses on a closer examination of the hMSC IN generated in Phase A (P+5) cultures utilizing their increased size and relative ease of sphere production.

### hMSC Induced Neurospheres Require HS for Formation

FDA/PI staining of the Phase A hMSC IN confirmed that the Phase A hMSC IN were comprised of a core of live cells with some dead cells distributed throughout the sphere (**Figure 3B**) and a relative intensity ratio of 3:1 (live to dead cells). HS epitope 10E4 staining identifying the *N*-sulfated glucosamine residues revealed the presence of high levels of HS in hMSC IN (**Figure 3C**).

### PG Biosynthesis Machinery: Initiation and Modification

Q-PCR analysis of PG biosynthesis machinery components (HSPG initiation and modification enzymes), along with HS GAG chain polymerization and sulfation enzymes were then examined in hMSC IN, undifferentiated hMSCs and embryonic stem cell-derived human neural stem cell H9 (hNSC H9) cultures, an established human neural culture model for comparison (Oikari et al., 2016b).

**FIGURE 4:**
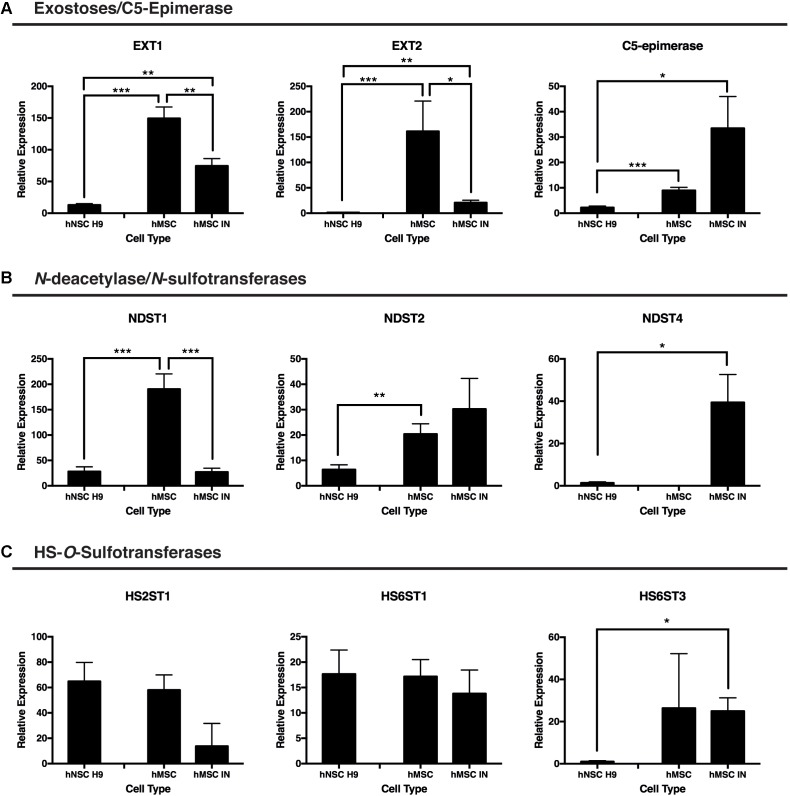
Gene expression changes for proteoglycan initiation and modification enzymes between induced and undifferentiated primary cultures. **(A)** Exostosins (EXT1/EXT2) are responsible for the polymerization of the growing glycosaminoglycan (GAG) chain and were significantly reduced in both human neural stem cells (hNSC H9) and human mesenchymal stem cell (hMSC) induced neurospheres (hMSC IN) when compared to undifferentiated hMSCs. In contrast, gene expression of C5-epimerase, responsible for epimerization of the GAG chain decreased in hNSC H9 cultures and increased in hMSC IN compared to hMSCs. **(B)** *N*-deacetylase/*N*-sulfotransferase (NDST) enzymes. Significantly reduced NDST1 gene expression was observed in hNSC H9 and hMSC IN compared to undifferentiated hMSCs; NDST2 demonstrated reduced expression in hNSC H9 cells. Interestingly, the neural specific NDST4 was significantly increased in hMSC IN when compared to hNSC H9s and was not detected in undifferentiated hMSC cultures. **(C)** Heparin sulfate specific *O*-sulfation enzymes showed little significant difference between the cell types with the only significant increase observed between hNSC H9s and hMSC IN for HS6ST3. Chondroitin sulfate specific *O*-sulfation enzymes displayed significantly reduced expression in hMSC IN compared to hNSC H9 and undifferentiated hMSC cultures. Significantly higher heparanase (HPSE) gene expression was detected in undifferentiated hMSCs compared to hNSC H9 cultures and non-significantly increased compared to hMSC IN. ^∗^*p* < 0.05, ^∗∗^*p* < 0.005, ^∗∗∗^*p* < 0.0001.

Significantly reduced gene expression of the GAG initiation enzymes EXT1 and EXT2 was observed in hMSC IN when compared with undifferentiated hMSC cultures (EXT1: *p* = 0.00097; EXT2: *p* = 0.02). Extremely high levels of these enzymes were detected in undifferentiated hMSC cultures when compared to hNSC H9 cultures (EXT1: *p* = 9.8 × 10^-10^; EXT2: *p* = 0.0098) and levels detected in hMSC IN also exceeded those detected in hMSC H9 for both enzymes (EXT1: *p* = 0.0006; EXT2: *p* = 0.004; **Figure 4A**).

C5-epimerase was detected at a significantly higher level in both undifferentiated hMSCs (*p* = 7.2 × 10^-6^) and hMSC IN (*p* = 0.04) when compared to the hNSC H9 cultures (**Figure 4A**). The increase in C5-epimerase expression in hMSC IN cultures did not reach significance when compared to undifferentiated hMSCs.

We observed significantly reduced expression of the ubiquitous *N*-sulfation enzyme, NDST1, in hMSC IN (*p* = 2.3 × 10^-6^) and in hNSC H9 (*p* = 3.2 × 10^-6^) cultures when compared to undifferentiated hMSC cultures (**Figure 4B**). Levels of NDST2 were observed to be significantly reduced in hNSC H9 cultures when compared to undifferentiated hMSCs (*p* = 0.003) while the neural specific NDST4 demonstrated significantly increased gene expression in hMSC IN when compared to hNSC H9 cultures (*p* = 0.03; **Figure 4B**). No gene expression of NDST4 was detected in undifferentiated hMSCs. HS6ST3 (sulfation at the 6-*O* position of the HS GAG chains) gene expression was also significantly increased in hMSC IN when compared to hNSC H9 cultures (*p* = 0.02; **Figure 4C**). No other significant differences were observed between cultures for the HS *O*-sulfation enzymes. Interestingly, these changes are similar to those seen when we previously compared hNSC H9 cultures to normal human neural progenitor (nhNPC; Oikari et al., 2016a) cells. This data suggests the processes converting hMSCs to hMSC IN are not dissimilar in HS requirements to the commitment of human neural stem cells to more lineage restricted neural progenitor cells.

### hMSC Induced Neurospheres Are Positive for Neural Progenitor Markers and HSPG Core Proteins

Gene expression and localisation of HSPG core proteins, SDC1, SDC4, GPC1 and GPC4 along with NSC self-renewal markers, Nestin and SOX2, and neural lineage specific markers, MAP2, TUBB3, S100B and GFAP were then examined in Phase A hMSC INs.

**FIGURE 5:**
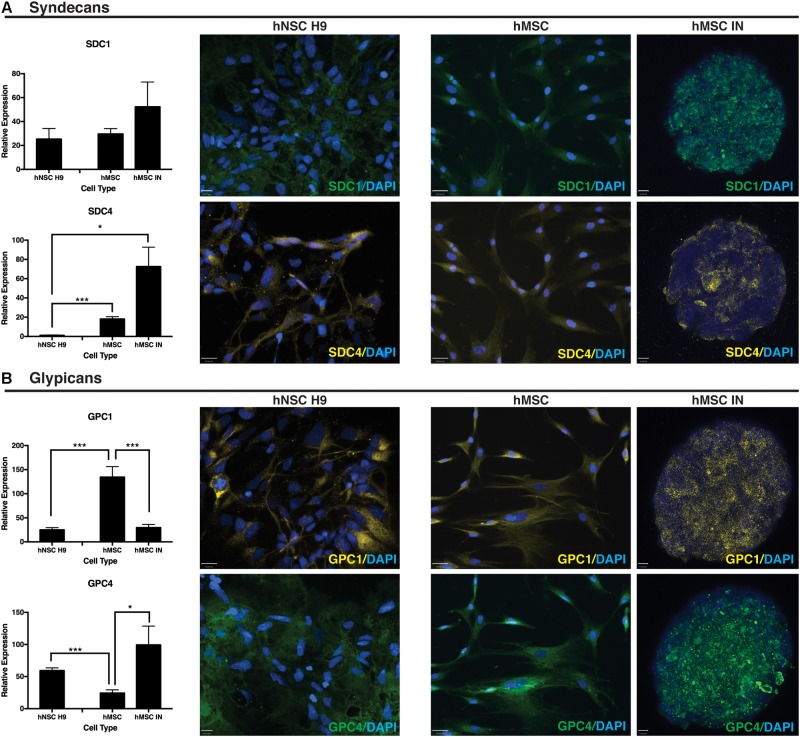
Heparin sulfate proteoglycan (HSPG) core proteins. **(A)** Syndecans. Representative immunocytochemistry (ICC) images from undifferentiated primary and induced cultures demonstrate no detectible visual difference in levels of syndecan 1 (SDC1) between culture conditions. This observation is supported by the corresponding Q-PCR data. Gene expression levels of syndecan 4 (SDC4) were significantly higher in hMSC IN compared to undifferentiated hMSCs and hNSC H9 cultures. Scale bars represent: hNSC H9 SDC1 70 μm, SDC4 100 μm; hMSC 100 μm; hMSC IN 16 μm. **(B)** Glypicans. Gene expression levels of glypican 1 (GPC1) were significantly higher in undifferentiated hMSC cultures than hNSC H9 and hMSC IN cultures. Similar levels of GPC1 were detected in the primary hNSC H9 cultures compared to hMSC IN. In contrast, significantly higher gene expression of glypican 4 (GPC4) was detected in the neural cultures (hNSC H9 and hMSC IN) when compared to undifferentiated hMSCs. ICC staining with primary antibodies specific to HSPG core proteins demonstrated visible differences between culture conditions, with this observation supported by Q-PCR results. Secondary antibodies: FITC (green) and Cy3 (yellow). Cultures counterstained with DAPI to reveal nuclei. Scale bars represent: hNSC H9 GPC1 100 μm, GPC4 70 μm; hMSC 100 μm; hMSC IN 10 μm. ^∗^*p* < 0.05, ^∗∗∗^*p* < 0.0001.

SDC1 gene expression remained unchanged when compared to undifferentiated and hNSC H9 cultures. SDC4 significantly increased in both undifferentiated hMSCs and hMSC IN when compared to hNSC H9 cultures (hMSC: *p* = 1.4 × 10^-8^; hMSC IN: *p* = 0.007; **Figure 5A**). Significantly reduced GPC1 was observed in hMSC IN (∼20%; *p* = 2.1 × 10^-5^) and hNSC H9 (*p* = 7.9 × 10^-6^) cultures when compared to undifferentiated hMSCs. Gene expression of GPC4 significantly increased in both hMSC IN (*p* = 0.03) and hNSC H9 (*p* = 9.1 × 10^-6^) cultures when compared to undifferentiated hMSCs (**Figure 5B**).

Positive ICC staining all HSPG core proteins examined (SDC1, SDC4, GPC1, GPC4) was observed in hNSC H9 and hMSC cultures. hNSC H9 staining revealed homogeneous, cytoplasmic staining with some distinct differences observed in undifferentiated hMSC and hMSC IN. Undifferentiated hMSC cultures revealed predominantly cytoplasmic staining for these proteins with GPC1 displaying distinct filamentous expression with expression of SDC1, SDC4 and GPC4 more diffuse. In hMSC IN cultures, SDC1, GPC1 and GPC4 demonstrated a similar staining pattern, with positively stained puncta detected across the majority of the cells within the sphere. In contrast, SDC4 demonstrated largely heterogeneous and more discrete staining with cells positive for this HSPG localized along the external edge and the inner core of the sphere (**Figure 5**).

**FIGURE 6:**
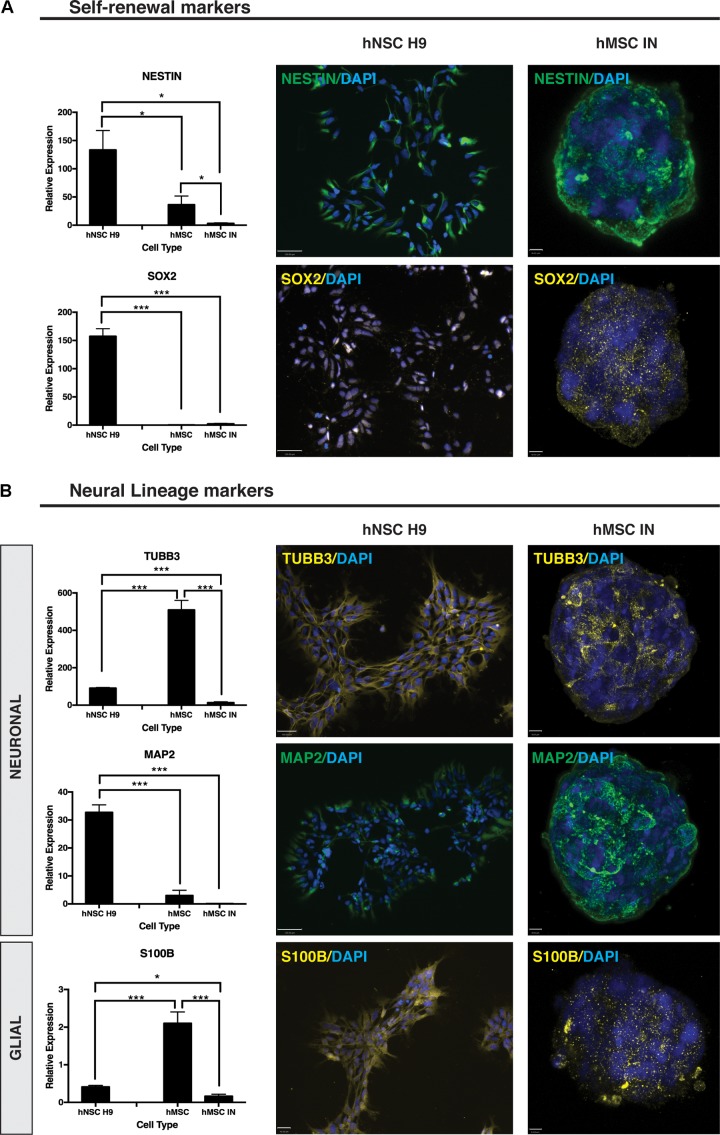
Neural self-renewal and lineage markers. **(A)** Self-renewal markers. Significantly reduced gene expression of neural stem cell self-renewal markers Nestin and SOX2 were detected in undifferentiated human mesenchymal stem cell (hMSC) and hMSC induced neurospheres (hMSC IN) when compared to human neural stem cell (hNSC H9) cultures. Staining in hMSC IN reveals visibly detectible expression of both these markers in hMSC IN cultures. Scale bars represent: hNSC H9 130 μm, hMSC IN 6 μm. **(B)** Neural Lineage Markers. Significantly higher gene expression levels of the early neuronal marker, βIII-tubulin (TUBB3), and the astrocyte marker (S100B) were detected in undifferentiated hMSC cultures compared to hNSC H9 and hMSC IN. Levels of both these genes were detected at significantly lower levels in hMSC IN than in the primary hNSC H9s. Levels of the late neuronal marker, MAP2, were significantly lower in both primary hMSC and induced hMSC IN cultures than in hNSC H9 cultures. Cultures were stained with specific primary antibodies for each marker of interest. Secondary antibodies: FITC (green) and Cy3 (yellow). Nuclei counterstained with DAPI (blue). Scale bars represent: hNSC H9 TUBB3 100 μm, MAP2 130 μm, S100B 70 μm; hMSC IN TUBB3 and MAP2 8 μm, S100B 6 μm. ^∗^*p* < 0.05, ^∗∗^*p* < 0.005, ^∗∗∗^*p* < 0.0001.

ICC staining of self-renewal markers Nestin and SOX2 revealed positive staining for both markers (**Figure 6A**) with Nestin demonstrating a uniform localization pattern (Okolicsanyi et al., 2015). The positive staining for this marker was similar to patterns seen in hNSC H9 cultures with distinct similarities in expression and localization. In contrast, SOX2 localization was distinctly cytoplasmic in hMSC IN cultures when compared to the nuclear localization observed in hNSC H9 cultures (**Figure 6A**). Gene expression analysis in hMSC IN identified significantly reduced expression of Nestin (*p* = 0.01) and SOX2 (*p* = 7.3 × 10^-6^) when compared with hNSC H9 cultures (**Figure 6A**). In addition, reduced expression of Nestin was observed in undifferentiated hMSCs when compared to hNSC H9 (*p* = 0.04), and in hMSC IN when compared to undifferentiated hMSC cultures (*p* = 0.04; **Figure 6A**). No significant difference in gene expression was observed for SOX2 between hMSC cultures, however, the expression of this gene was significantly lower in undifferentiated hMSCs when compared to hNSC H9 cultures (*p* = 7.6 × 10^-6^; **Figure 6A**). Several additional significant changes in gene expression of neural markers Nanog homeobox (NANOG; pluripotency) and ENO2 (neuronal) were also observed. Expression of NANOG was significantly increased in hMSC IN when compared to undifferentiated hMSCs (*p* = 0.03), similar to observations in more lineage restricted nhNPC cultures (Oikari et al., 2016a), and significantly reduced when compared to hNSC H9 (*p* = 0.02). The expression level of NANOG in undifferentiated hMSC cultures was significantly lower than in hNSC H9 cultures (*p* = 0.007; **Supplementary Figure S4A**). Gene expression levels of ENO2 were significantly increased in hMSC IN when compared to both undifferentiated hMSCs (*p* = 0.02) and hNSC H9 (*p* = 0.02) (**Supplementary Figure S4B**). The early neuronal marker TUBB3 was significantly reduced in hMSC IN when compared to both undifferentiated hMSC (*p* = 5.1 × 10^-13^) and hNSC H9 (*p* = 1.1 × 10^-9^) cultures. However, in undifferentiated hMSCs TUBB3 expression was significantly higher than in hNSC H9 cultures (*p* = 2.3 × 10^-11^; **Figure 6B**). In addition, ICC analysis revealed distinct TUBB3 staining in hMSC IN, with approximately 50% cells staining positive throughout the sphere (**Figure 6B**). The late neuronal marker, MAP2, was also examined using both ICC and Q-PCR. While gene expression levels were significantly reduced in both hMSCs (*p* = 3.7 × 10^-7^) and hMSC IN (*p* = 5.7 × 10^-6^) when compared to hNSC H9, strong staining of this marker in pattern and intensity similar to the staining for Nestin was observed within the hMSC IN (**Figure 6B**) when compared with <2% of the hNSCH9 cells staining positive (Okolicsanyi et al., 2015).

S100B demonstrated heterogeneous diffuse and punctate staining within hMSC IN (**Figure 6B**) with significantly reduced expression in both hNSC H9 (*p* = 9.2 × 10^-6^) and hMSC IN (*p* = 1.2 × 10^-6^) when compared to undifferentiated hMSC cultures (**Figure 6B**). In addition, levels of S100B detected in hMSC IN were significantly reduced when compared to hNSC H9 cultures (*p* = 0.02; **Figure 6B**). An additional glial marker, GALC, was detected at significantly lower levels in hNSC H9 than both hMSC (*p* = 0.007) and hMSC IN (*p* = 0.0002). Levels of GALC were also significantly lower in hMSCs than in hMSC IN (*p* = 0.0004). Levels of CD44 were significantly lower in hNSC H9 cultures than both hMSC (4.93 × 10e5) and hMSC IN (*p* = 0.01) cultures (**Supplementary Figure S4C**).

In addition to the gene expression changes observed for HSPGs, stemness and neural lineage markers, there was a significant reduction in mesenchymal marker expression including: Smooth muscle actin 2 (ACTA2; *p* = 1.2401 × 10^-8^), Alkaline Phosphatase (AP: *p* = 2 × 10^-9^), Adipose-Q (ADIPO-Q; *p* = 1.4 × 10^-5^), Collagen 1A1 (COL1A1; *p* = 1.2 × 10^-9^) and Peroxisome proliferator-activated receptor gamma 1 (PPARG1; *p* = 0.02) when compared to undifferentiated hMSC cultures (**Supplementary Figure S5**). This data further supports the increased neural potential of hMSC IN and subsequent reduced mesenchymal lineage potential following sphere formation and exposure to neuronal lineage culture conditions.

## Discussion

The inherent heterogeneity of stem cell cultures likely provides the key to their efficacy in therapeutic applications. MSCs have the potential to repair developmental and bone defects, along with neurodegenerative injuries and disorders. Here, we investigated HSPGs for their role in neural lineage differentiation of hMSCs with these cells previously identified to express a number of markers of neural stemness and the neural lineages (neuronal, astrocyte, oligodendrocyte) throughout *in vitro* expansion (Okolicsanyi et al., 2015). hMSCs were shown to utilize HSPGs *in vitro* in basal culture conditions with an increase in cell number following addition of heparin to the culture media. In addition, the expression of neural markers was shown to be responsive to these changes in the *in vitro* niche with altered expression and localization following modifications mediated by HSPGs correlating to or complementing our observed changes in HSPG expression. Although the observed changes in expression were not uniform across growth phases, this data suggests that particular marker combinations could be used to direct lineage specification potentially enabling the production of increased, lineage-specific neural MSC-derived cultures.

To examine the early stages of neural commitment, we generated and examined hMSC IN cultures. Sphere formation efficiency varied between populations and growth phases, however, consistent low levels of expression were observed for neural lineage markers (astrocyte, neuronal, oligodendrocyte). Similarities in expression patterns between HSPG core proteins and neural markers in undifferentiated hMSCs indicate the involvement of these proteins in the maintenance of neural lineage potential in MSCs. Interestingly, the combined expression and localization of HSPGs with several neural self-renewal markers (Nestin, SOX2) along with the maintained expression and localization profile during sphere formation of an intermediary neurogenic cell type, suggest the use of hMSC cultures to generate hMSC IN could have a considerable impact on their use in therapeutic applications. The key involvement of HSPGs during neural specification of these cells and the identification of HSPGs in hMSC IN formation correlate with the reported roles of HSPGs during neural development.

The lack of significant changes in the common initiation and polymerization enzymes during neural specification suggests a requirement for GAG chains in the conversion from undifferentiated hMSCs to neural lineages. In addition, we would not expect to see complete loss of the polymerization enzymes (EXTs) as both EXTs are required to form a complex for significant polymerization of the GAG chains (McCormick et al., 2000). However, it is likely that each cell type requires varying levels and efficiency of EXT activity in response to local cellular cues. Evidence presented here supports this, with the ratio of EXT1:EXT2 maintained between all culture conditions (higher EXT1 compared to EXT2). In addition, levels of EXT2 were reduced in nhNPCs when compared to hNSC H9s (Oikari et al., 2016a) demonstrating a similarity between the more lineage restricted cell types (hMSC IN and nhNPCs).

A family of four *N*-deacetylase/*N*-sulfotransferases, the NDSTs, perform *N*-sulfation, critical for the production of HS GAG chains. Levels of the ubiquitous *N*-sulfation enzymes were decreased in hMSC IN when compared with undifferentiated hMSCs with NDST1 demonstrating a significant decrease in expression suggesting that neural specification entails altered N-deacetylase/*N*-sulfotransferase activity. Interestingly, significantly increased levels of NDST4 in hMSC IN suggest an increase in neural specific HSPGs with NDST4 predominantly found *in vivo* in the adult brain (Aikawa et al., 2001; Grobe et al., 2002). NDST1 and NDST2 remained at detectable levels in hMSC IN, supporting expression of NDST1 and NDST2 during induction of neural cells (Forsberg et al., 2012). The elevated levels of the NDSTs observed in hMSC IN correlate with the elevated level of C5-epimerase, with epimerization of the growing GAG chain reliant upon *N*-sulfation to enable further *O*-sulfation of the growing GAG chain (Kim et al., 2001; Sugahara and Kitagawa, 2002). Our previous data demonstrated significantly increased C5-epimerase expression in nhNPCs when compared with hNSC H9s (Oikari et al., 2016a), linking this enzyme with neural lineage commitment. The influence of active HSPG biosynthesis machinery during the conversion of hMSCs to hMSC IN is further supported by numerous studies demonstrating specific *O*-sulfation patterns determine and facilitate interactions with individual growth factors and morphogens to drive specialized cellular processes (Sugahara and Kitagawa, 2002; Habuchi et al., 2004; Johnson et al., 2007).

With the specificity of interactions between HSPGs and ligands dependent on specific combinations of GAG chain length and sulfation pattern, any alteration in length or composition directly impacts (to enhance or inhibit) interactions with specific signaling partners. Changes in HSPG modification enzymes in this study suggest remodeling of the hMSC IN microenvironment during sphere formation and maintenance along with a key role for HSPGs in neural lineage differentiation of hMSCs. The observed expression changes in HS biosynthesis enzymes in hMSC IN when compared to hNSC H9 cultures, provide consistency with this model, with previous comparisons between hNSC and hNPC supporting similar changes in cultures following sphere formation (Oikari et al., 2016a).

Although some inconsistency is exhibited by the gene expression changes identified by Q-PCR, correlations with staining of HSPGs in hMSC IN support potential differential functions for HSPGs during neural specification. The HSPG core protein SDCs and GPCs, have been identified to have neural lineage and differentiation stage specific expression (Oikari et al., 2016b). A role for SDC1 in maintaining stemness within the hMSC IN cell population is indicated by the observed increase in SDC1 expression following hMSC IN, supporting previous work from our group demonstrating lower SDC1 gene expression in neural progenitor cells (nhNPCs) when compared with hNSC H9s (Oikari et al., 2016a). The dual role of SDC4 has previously been identified by increased cellular adhesion in a human breast cancer model (Lendorf et al., 2011) and increased cellular communication and signaling (Dwyer and Esko, 2016) through decreased motility in the neural microenvironment. As cells progress toward a terminally differentiated state, characterized by a lack of motility, our previous data show increased levels of SDC4 in nhNPCs when compared with hNSC H9s (Oikari et al., 2016a) supporting this role for SDC4 in neural lineage specification. In this study, significantly elevated SDC4 levels in undifferentiated hMSCs and hMSC IN compared to hNSC H9 cultures further reflect the more lineage-restricted capacity of hMSC IN than parental hMSC or hNSC H9s cultures (Oikari et al., 2016a).

The gene expression and localization of GPC1 implicate a key role for this HSPG in neuronal lineage specification, but also reinforce clear differences between hMSC IN and the neural progenitor cultures (Oikari et al., 2016a). In addition, in the more lineage-restricted nhNPCs significantly decreased levels of GPC4 may indicate a role for this HSPG in maintaining stemness in these cells (Oikari et al., 2016a). Interestingly, the significantly increased GPC4 observed in hMSC IN when compared to the basal monolayer hMSC cultures, may indicated de-differentiation or an increase in stemness of hMSC IN, providing further evidence that these cultures represent an intermediary neurogenic cell type.

## Conclusion

**FIGURE 7:**
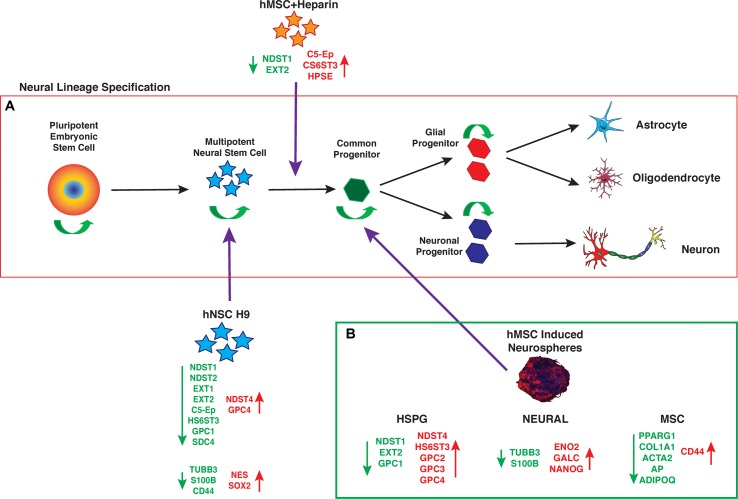
Neural lineage specification. **(A)** Established progression of maturing cells during neural lineage specification from pluripotent embryonic stem cells through to specified, mature neural cell types. As cells mature they retain some self-renewal ability (green arrows) while becoming more lineage restricted, until the final stages of specification when cells differentiate to produce a predetermined mature cell type with specific functions. **(B)** Summary of gene expression changes seen in human mesenchymal stem cell (hMSC) induced neurospheres (hMSC IN), relative to undifferentiated hMSCs. Note the similarities in gene expression changes (heparan sulfate proteoglycan; HSPG and neural) between the hMSC IN and human neural stem cells (hNSC H9). In addition, the summary highlights the down regulation of mesenchymal specific markers used to identify the traditional mesodermal lineage potential of hMSCs. This data provides supporting evidence that generation of hMSC IN cultures results in a more lineage restricted cell type than a true neural stem cell, but that this common progenitor-like cell retains multi-lineage capacity, with increased neural potential. (Mature cell type images from: Ivins et al., 1997; Johnson et al., 2007).

The data presented here support hMSC IN derived from human bone marrow MSCs generate a more lineage-restricted common neural progenitor-like population to undifferentiated hMSC and hNSC H9 cultures. The observed changes in neural stemness and lineage specific markers are not conclusive of terminal lineage specification and functionality, however, their maintained expression indicate hMSC IN retain these key functional attributes in a similar fashion to true neural progenitor cells (**Figure 7**). HSPG regulation of hMSCs neural lineage specification may occur through direct interaction or through the sequestration or presentation of appropriate growth factors, both mediated by the cellular microenvironment and key HSPGs. This needs to be further investigated along with a more detailed analysis of the structural contribution of the cultures i.e., spheres vs. monolayer during neural specification *in vitro* and *in vivo*. hMSCs may provide an abundant source of cells that can be manipulated via HSPGs and maintained *in vitro* for neural repair and regeneration and have potential in therapeutic applications following neurological trauma or disease such as Dementias.

## Author Contributions

RO conceived and designed the study, collected/assembled the data, performed data analysis and interpretation, and drafted and finalized the manuscript. LO collected/assembled data, performed data analysis and interpretation, and contributed to the writing of the manuscript. CY collected/assembled the data and contributed to the writing of the manuscript. LG provided financial support and contributed to the editing and finalization of the manuscript. LH conceived of the study and experimental design, provided financial support and provision of materials, and contributed to data analysis and interpretation, writing, editing, and finalization of the manuscript.

## Conflict of Interest Statement

The authors declare that the research was conducted in the absence of any commercial or financial relationships that could be construed as a potential conflict of interest.

## Supplementary Material

FIGURE S1 Human mesenchymal stem cell (hMSC) dose response. Average cell numbers of hMSCs following treatment with 0, 1, 5, 10, 25, and 50 μg/mL of heparin over 1 (D1), 3 (D3) or 5 days (D5). Average cell numbers of hMSCs following treatment with 0, 1, 5, 50, 100 and 500 mM sodium chlorate over D1, D3, or D5. Cell numbers are averages collected from three hMSC populations with experiments conducted in triplicate (*n* = 9). Data is presented ± SEM. 10 μg/mL was selected as the optimal concentration of heparin for further experiments, while 50 mM sodium chlorate was selected for further investigation.

FIGURE S2 Gene expression of heparan sulfate proteoglycan (HSPG) core proteins at each distinct growth phase (Phase A-C). **(A)** Syndecans (SDC). SDC1-3 expression increases throughout *in vitro* expansion. SDC1 is consistently detected at levels approximately 30–50% of SDC3. SDC2 levels are consistently detected at approximately 50% of SDC3. In contrast to SDC1-3, SDC4 levels are maintained throughout *in vitro* expansion with levels observed approximately 50% of SDC1 at growth phase A. **(B)** Glypicans (GPC). Gene expression of GPC1 increased throughout *in vitro* expansion with the greatest increase in expression observed between Phase B and Phase C of growth. GPC4 and GPC6 were also detected consistently throughout *in vitro* expansion with levels of both these genes remaining below 50% of GPC1 at Phase A. GPC2 and GPC3 were detected at levels approximately 1–5% of GPC1 Phase A levels, however, neither of these genes were detected at growth phase A.

FIGURE S3 Neural response to niche modification. **(A)** Glial markers. GalC expression generally decreased after treatment with heparin, except at Phase B where a non-significant increase in expression was observed. Heparin treatment generally resulted in non-significantly increased gene expression of Olig2, except at Phase A where a non-significant decrease was observed. **(B)** The additional neuronal markers examined showed an overall decreased gene expression following treatment of cultures with heparin, with the exception of NCAD at Phase A where a non-significant increase in expression was observed. Significantly decreased gene expression of NCAD was observed at Phase C and Phase A and B for TUBB3. ^∗^*p* < 0.05, ^∗∗^*p* < 0.005, ^∗∗∗^*p* < 0.0001.

FIGURE S4 Additional Neural Self-renewal and Neural lineage markers. **(A)** Pluripotency marker, NANOG. Levels of NANOG detected in primary hMSCs was significantly lower than in both hMSC IN and hNSC H9 cultures. Gene expression levels of NANOG were also significantly lower in hMSC IN than in hNSC H9 cultures. **(B)** Neuronal markers. Levels of ENO2 were significantly lower in both primary cultures, hNSC H9 and hMSC compared to hMSC IN. Levels of NEFM were significantly higher in undifferentiated hMSCs compared to hNSC H9. There were no significant differences in NEFM expression between hMSC IN and hMSCs. **(C)** Glial markers. GALC was detected at significantly lower levels in hNSC H9 than both hMSC and hMSC IN. Levels of GALC were also significantly lower in hMSCs than in hMSC IN. Levels of CD44 were significantly lower in hNSC H9 cultures than both hMSC and hMSC IN cultures. ^∗^*p* < 0.05, ^∗∗^*p* < 0.005, ^∗∗∗^*p* < 0.0001.

FIGURE S5 Mesenchymal lineage markers. Levels of mesenchymal lineage markers Smooth muscle actin 2(ACTA2), Alkaline Phosphatase (AP), Adipose –Q (ADIPOQ), Collagen 1A1 (COL1A1), Peroxisome proliferator-activated receptor gamma 1 (PPARG1) were significantly lower in hMSC IN than in undifferentiated hMSC cultures. ^∗^*p* < 0.05, ^∗∗^*p* < 0.005, ^∗∗∗^*p* < 0.0001.

TABLE S1 Primer sequences used for Q-PCR analysis.
