# Supplementary figures and images for: Heparan Sulfate Proteoglycans as Drivers of Neural Progenitors Derived From Human Mesenchymal Stem Cells
Source: Front Mol Neurosci. 2018 Apr 24;11:134. doi: 10.3389/fnmol.2018.00134 (PMC5928449; doi:10.3389/fnmol.2018.00134)

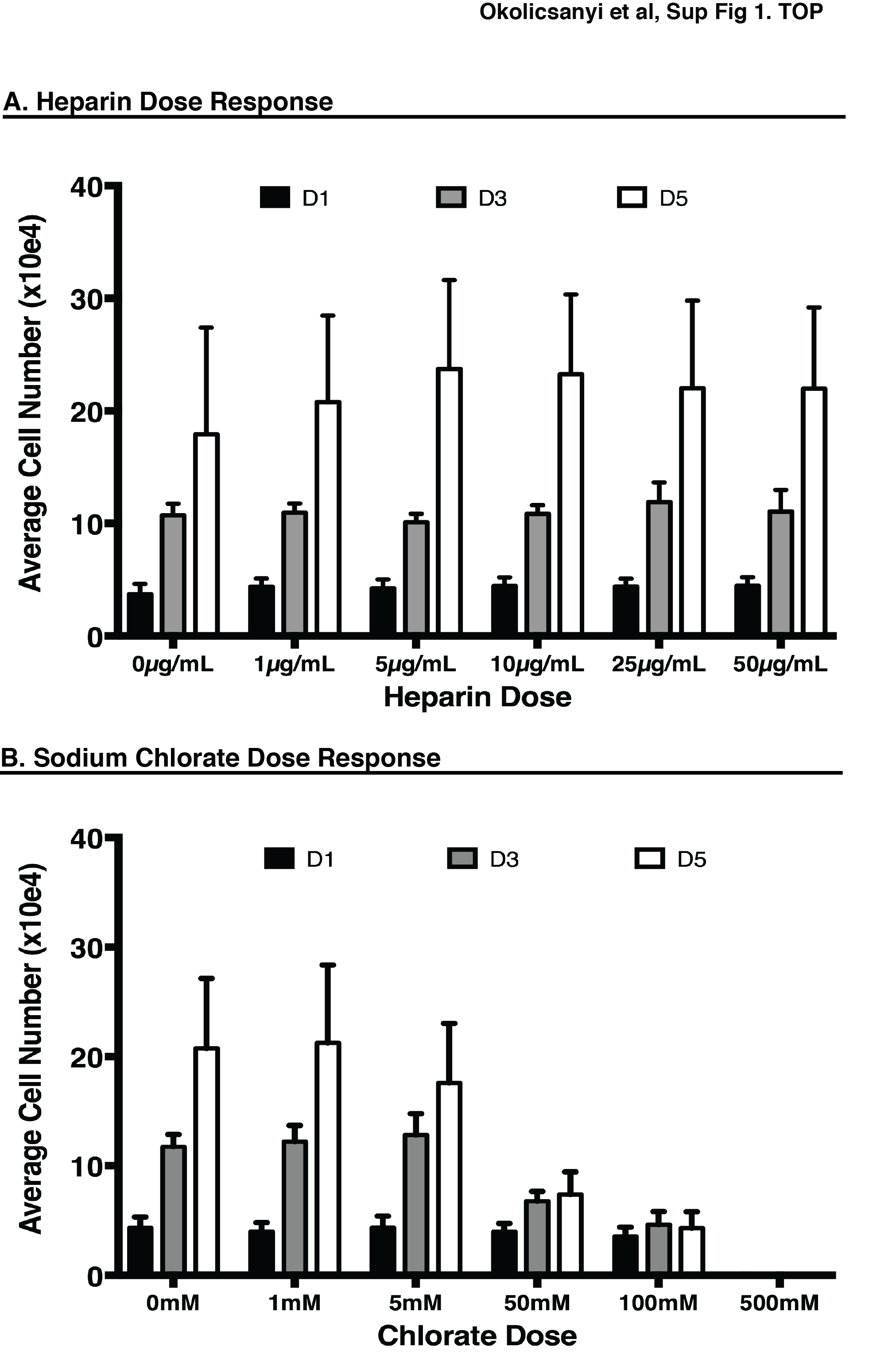

Supplement: FIGURE S1 — Human mesenchymal stem cell (hMSC) dose response. Average cell numbers of hMSCs following treatment with 0, 1, 5, 10, 25, and 50 μg/mL of heparin over 1 (D1), 3 (D3) or 5 days (D5). Average cell numbers of hMSCs following treatment with 0, 1, 5, 50, 100 and 500 mM sodium chlorate over D1, D3, or D5. Cell numbers are averages collected from three hMSC populations with experiments conducted in triplicate (n = 9). Data is presented ± SEM. 10 μg/mL was selected as the optimal concentration of heparin for further experiments, while 50 mM sodium chlorate was selected for further investigation. [file Image_1.tif]

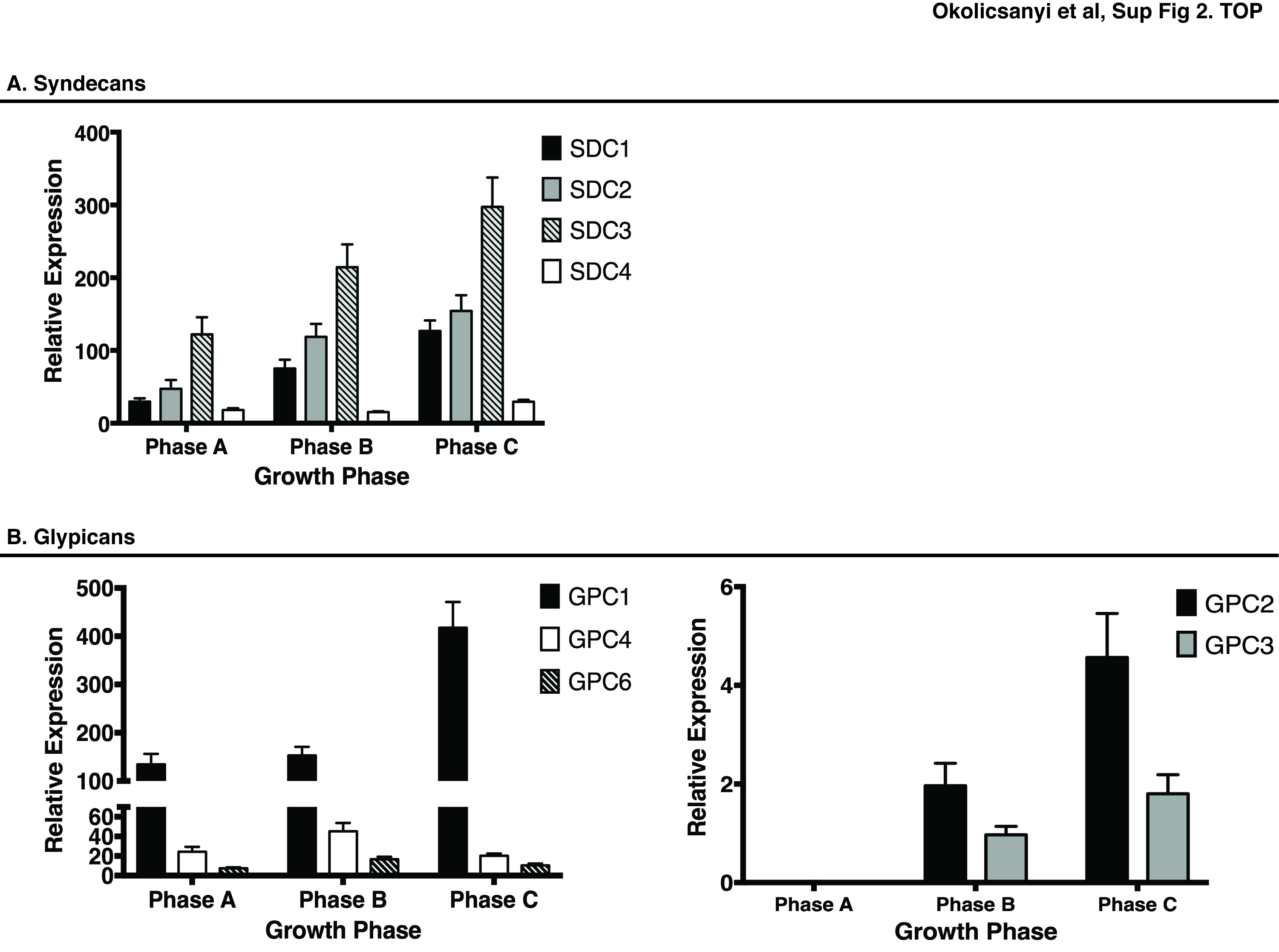

Supplement: FIGURE S2 — Gene expression of heparan sulfate proteoglycan (HSPG) core proteins at each distinct growth phase (Phase A-C). (A) Syndecans (SDC). SDC1-3 expression increases throughout in vitro expansion. SDC1 is consistently detected at levels approximately 30–50% of SDC3. SDC2 levels are consistently detected at approximately 50% of SDC3. In contrast to SDC1-3, SDC4 levels are maintained throughout in vitro expansion with levels observed approximately 50% of SDC1 at growth phase A. (B) Glypicans (GPC). Gene expression of GPC1 increased throughout in vitro expansion with the greatest increase in expression observed between Phase B and Phase C of growth. GPC4 and GPC6 were also detected consistently throughout in vitro expansion with levels of both these genes remaining below 50% of GPC1 at Phase A. GPC2 and GPC3 were detected at levels approximately 1–5% of GPC1 Phase A levels, however, neither of these genes were detected at growth phase A. [file Image_2.TIF]

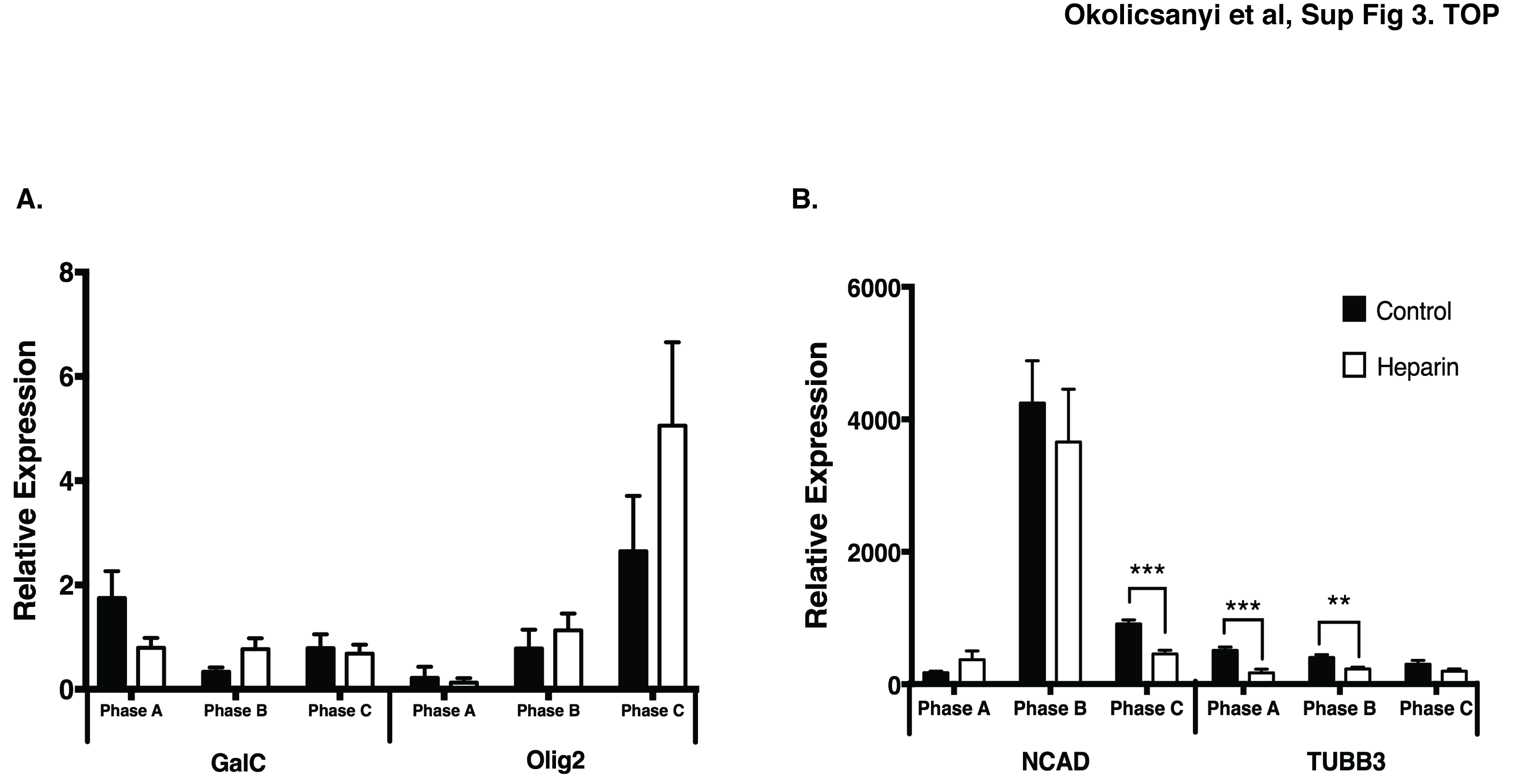

Supplement: FIGURE S3 — Neural response to niche modification. (A) Glial markers. GalC expression generally decreased after treatment with heparin, except at Phase B where a non-significant increase in expression was observed. Heparin treatment generally resulted in non-significantly increased gene expression of Olig2, except at Phase A where a non-significant decrease was observed. (B) The additional neuronal markers examined showed an overall decreased gene expression following treatment of cultures with heparin, with the exception of NCAD at Phase A where a non-significant increase in expression was observed. Significantly decreased gene expression of NCAD was observed at Phase C and Phase A and B for TUBB3. ∗p < 0.05, ∗∗p < 0.005, ∗∗∗p < 0.0001. [file Image_3.TIF]

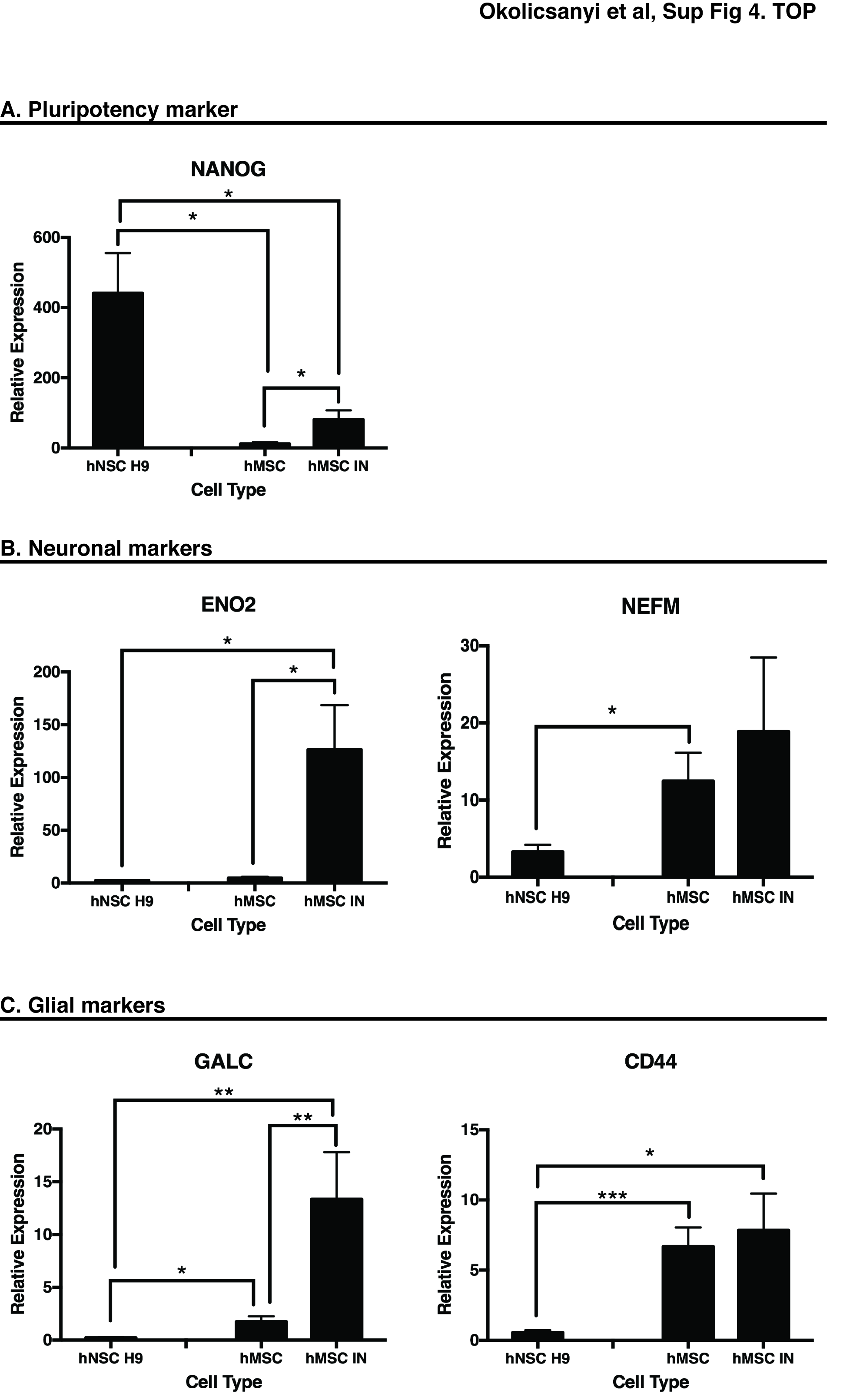

Supplement: FIGURE S4 — Additional Neural Self-renewal and Neural lineage markers. (A) Pluripotency marker, NANOG. Levels of NANOG detected in primary hMSCs was significantly lower than in both hMSC IN and hNSC H9 cultures. Gene expression levels of NANOG were also significantly lower in hMSC IN than in hNSC H9 cultures. (B) Neuronal markers. Levels of ENO2 were significantly lower in both primary cultures, hNSC H9 and hMSC compared to hMSC IN. Levels of NEFM were significantly higher in undifferentiated hMSCs compared to hNSC H9. There were no significant differences in NEFM expression between hMSC IN and hMSCs. (C) Glial markers. GALC was detected at significantly lower levels in hNSC H9 than both hMSC and hMSC IN. Levels of GALC were also significantly lower in hMSCs than in hMSC IN. Levels of CD44 were significantly lower in hNSC H9 cultures than both hMSC and hMSC IN cultures. ∗p < 0.05, ∗∗p < 0.005, ∗∗∗p < 0.0001. [file Image_4.TIF]

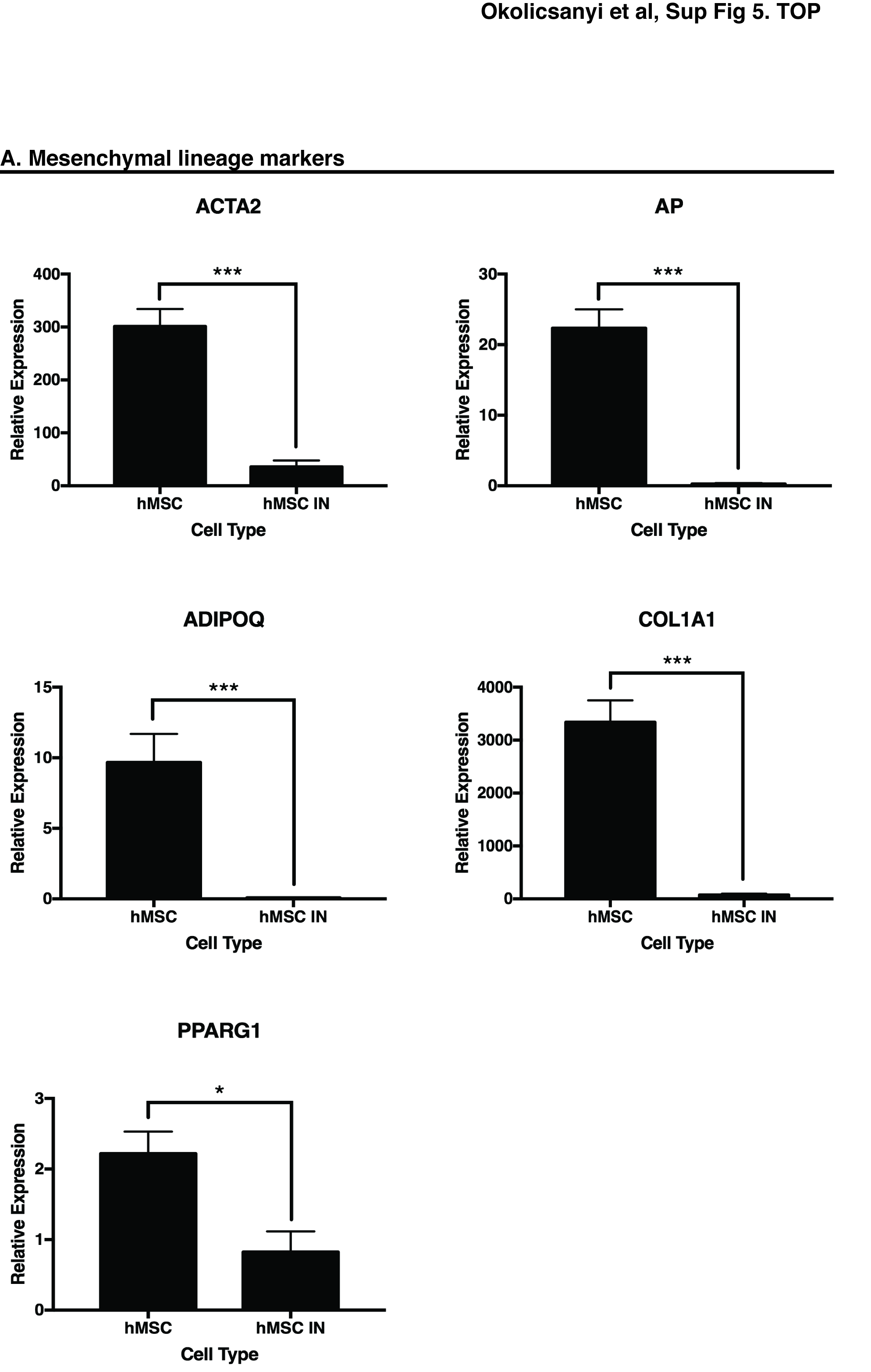

Supplement: FIGURE S5 — Mesenchymal lineage markers. Levels of mesenchymal lineage markers Smooth muscle actin 2(ACTA2), Alkaline Phosphatase (AP), Adipose –Q (ADIPOQ), Collagen 1A1 (COL1A1), Peroxisome proliferator-activated receptor gamma 1 (PPARG1) were significantly lower in hMSC IN than in undifferentiated hMSC cultures. ∗p < 0.05, ∗∗p < 0.005, ∗∗∗p < 0.0001. [file Image_5.TIF]
