# Supplementary material for: Heparan Sulfate Proteoglycans as Drivers of Neural Progenitors Derived From Human Mesenchymal Stem Cells
Source: Front Mol Neurosci. 2018 Apr 24;11:134. doi: 10.3389/fnmol.2018.00134 (PMC5928449; doi:10.3389/fnmol.2018.00134)

Supplementary Table 1. Primer Sequences for Q-PCR

| **Gene Name** | **Gene Symbol** | **Forward Primer** | **Reverse Primer** | **Amplicon Size** | **Ref Seq** | |
| --- | --- | --- | --- | --- | --- | --- |
| **Initiation and *N*-sulfation Enzymes** | | | | | |  |
| **N-deacetylase/** | NDST1 | TGGTCTTGGATGGCAAACTG | CGCCAAGGTTTTGTGGTAGTC | 107 | NM_001543 | |
| **N-sulfotransferase 1** |  |  |  |  |  |  |
| **N-deacetylase/** | NDST2 | CCTATTTGAAAAAAGTGCCACCTACT | GCAGGGTTGGTGAGCACTGT | 105 | NM_003635 | |
| **N-sulfotransferase 2** |  |  |  |  |  |  |
| **N-deacetylase/** | NDST3 | ACCCTTCAGACCGAGCATACTC | CCCGGGACCAAACATCTCTT | 151 | NM_004784 | |
| **N-sulfotransferase 3** |  |  |  |  |  |  |
| **N-deacetylase/** | NDST4 | ATAAAGCCAATGAGAACAGCTTACC | GGTAATATGCAGCAAAGGAGATTGA | 122 | NM_022569 | |
| **N-sulfotransferase 4** |  |  |  |  |  |  |
| **Exotosin-1** | EXT1 | TGACAGAGACAACACCGAGTATGA | GCAAAGCCTCCAGGAATCTGAAG | 119 | NM_000127.2 | |
| **Exotosin-2** | EXT2 | CAGTCAATTAAAGCCATTGCCCTG | GGGATCAGCGGGAGGAAGAG | 149 | NM_000401 | |
| **C5-Epimerase** | C5-Ep | AGCTGTCAAGCCAACCAAAATAA | CTTACTAGCCAATCACTAGCAGCAA | 138 | AY635582 | |
| **HS and CS *O*-Sulfation Enzymes and HS Cleavage Enzymes** | | | | | |  |
| **Heparan sulfate 2-O-sulfotransferase 1** | HST2ST1 | TCCCGCTCGAAGCTAGAAAG | CGAGGGCCATCCATTGTATG | 80 | NM_012262 | |
| **Heparan sulfate 6-O-sulfotransferase 1** | HS6ST1 | AGCGGACGTTCAACCTCAAGT | GCGTAGTCGTACAGCTGCATGT | 139 | NM_004807 | |
| **Heparan sulfate 6-O-sulfotransferase 3** | HS6ST3 | ACATCACGCGGGCTTCTAACGT | GGCGGTCCCTCTGGTGCTCTA | 156 | NM_153456 | |
| **Syndecans** | | | | | |  |
| **Syndecan-1** | SDC1 | CTGGGCTGGAATCAGGAATATTT | CCCATTGGATTAAGTAGAGTTTTGC | 76 | BC008765.2 | |
| **Syndecan-2** | SDC2 | AGCTGACAACATCTCGACCACTT | GCGTCGTGGTTTCCACTTTT | 72 | NM_002998.3 | |
| **Syndecan-3** | SDC3 | CTTGGTCACACTGCTCATCTATCG | GCATAGAACTCCTCCTGCTTGTC | 118 | AF248634 | |
| **Syndecan-4** | SDC4 | CCACGTTTCTAGAGGCGTCACT | CTGTCCAACAGATGGACATGCT | 76 | BC030805.1 | |
| **Glypicans** | | | | | |  |
| **Glypican-1** | GPC1 | GGACATCACCAAGCCGGACAT | GTCCACGTCGTTGCCGTTGT | 100 | NM_002081 | |
| **Glypican-2** | GPC2 | TGATCAGCCCCAACAGAGAAA | CCACTTCCAACTTCCTTCAAACC | 75 | NM_152742 | |
| **Glypican-3** | GPC3 | GATACAGCCAAAAGGCAGCAA | GCCCTTCATTTTCAGCTCATG | 71 | NM_004484. | |
| **Glypican-4** | GPC4 | GGTGAACTCCCAGTACCACTTTACA | GCTTCAGCTGCTCCGTATACTTG | 71 | NM_001448 | |
| **Glypican-6** | GPC6 | CAGCCTGTGTTAAGCTGAGGTTT | GATGTGTGTGCGTGGAGGTATGT | 71 | NM_005708. | |
| **Neural Markers** | | | | | |  |
| **Enolase 2** | ENO2 | TGCACAGGCCAGATCAAGAC | ACAGCACACTGGGATTACGG | 139 | [NM_001975.2](http://www.ncbi.nlm.nih.gov/nucleotide/16507966?report=gbwithparts) | |
| **Glial Fibrilic Acidic Protein** | GFAP | GCGGGATGGAGAGGTCATTA | CAGAGGCGGAGCAACTATCC | 131 | [NM_002055.4](http://www.ncbi.nlm.nih.gov/nucleotide/334688841?report=gbwithparts) | |
| **Microtubule Associated Protein 2** | MAP2 | GACTGCAGCTCTGCCTTTAG | AAGTAAATCTTCCTCCACTGTGAC | 106 | [NM_002374.3](http://www.ncbi.nlm.nih.gov/nucleotide/87578395?report=gbwithparts) | |
| **Nanog homeobox** | NANOG | ACCTCAGCTACAAACAGGTGAA | AAAGGCTGGGGTAGGTAGGT | 135 | [NM_024865.2](http://www.ncbi.nlm.nih.gov/nucleotide/153945815?report=gbwithparts) | |
| **Neural Filament Medium Chain** | NEFM | TGCAGTCCAAGAGCATCGAG | GGATGGTGTCCTGGTAGCTG | 120 | [NM_005382.2](http://www.ncbi.nlm.nih.gov/nucleotide/157738648?report=gbwithparts) | |
| **Nestin** | NES | CTCAGCTTTCAGGACCCCAA | GTCTCAAGGGTAGCAGGCAA | 128 | [NM_006617.1](http://www.ncbi.nlm.nih.gov/nucleotide/38176299?report=gbwithparts) | |
| **POU Class 5 homeobox 1** | OCT3/4 | ATCTTCAGGAGATATGCAAAGCAGA | TGATCTGCTGCAGTGTGGGT | 135 | [NM_002701.4](http://www.ncbi.nlm.nih.gov/nucleotide/116235483?report=gbwithparts) | |
| **SRY (sex determining region Y) box 2** | SOX2 | CCACCTACAGCATGTCCTACTCG | GGGAGGAAGAGGTAACCACAGG | 117 | NM_003106.3 | |
| **B III Tubbulin** | TUBB3 | GGCCAAGTTCTGGGAAGTCAT | CTCGAGGCACGTACTTGTGA | 137 | [NM_006086.3](http://www.ncbi.nlm.nih.gov/nucleotide/308235961?report=gbwithparts) | |
| **Oligodendrocyte transcription factor 2** | Olig2 | GACAAGCTAGGAGGCAGTGG | CGGCTCTGTCATTTGCTTCT | 111 | **NM_005806.3** | |
| **Galactosylceramidase** | GalC | GCCAAGCGTTACCATGATTT | TTTCACTCGCTGGAGACCTT | 123 | **NM_001201402.1** | |
| **S100 Calcium binding protein B** | S100B | TTCTGGAAGGGAGGGAGACA | CTCCTGCTCTTTGATTTCCTCT | 103 | **NM_006272.2** | |
| **Neural Cadherin** | NCAD | GAGGGATCAAAGCCTGGAACA | TTGAGGGCATTGGGATCGTC | 71 | [NM_001792.3](http://www.ncbi.nlm.nih.gov/entrez/viewer.fcgi?db=nucleotide&id=215422305) | |
| **Mesenchymal Markers** | | | | | |  |
| **Smooth Muscle Actin2** | ACTA2 | TAAGACGGGAATCCTGTGAAGC | TACAGAGCCCAGAGCCATTG | 90 | NM_001141945.1 | |
| **Adipose-Q** | ADIPO-Q | AAAGGAGATCCAGGTCTTATTGGT | GTTCTCCTTTCCTGCCTTGGA | 109 | [NM_001177800.1](http://www.ncbi.nlm.nih.gov/nucleotide/295317371?report=gbwithparts) | |
| **Hyaluronin Receptor** | CD44 | AGCAACTGAGACAGCAACCA | AGACGTACCAGCCATTTGTGT | 115 | [NM_000610.3](http://www.ncbi.nlm.nih.gov/nucleotide/48255934?report=gbwithparts) | |
| **Colagen 1A1** | COL1A1 | ACATGTTCAGCTTTGTGGACC | TGATTGGTGGGATGTCTTCGT | 117 | [NM_000088.3](http://www.ncbi.nlm.nih.gov/nucleotide/110349771?report=gbwithparts) | |
| **Alkaline Phosphatase** | AP | ATGCCCTGGAGCTTCAGAAG | TGGTGGAGCTGACCCTTGAG | 121 | NM_000478 | |
| **Peroxisome proliferator-activated receptor gamma 1** | PPARG1 | GGCCGCAGATTTGAAAGAAG | ATTTCGTTAAAGGCTGACTCTCGTT | 86 | NM_138712 | |


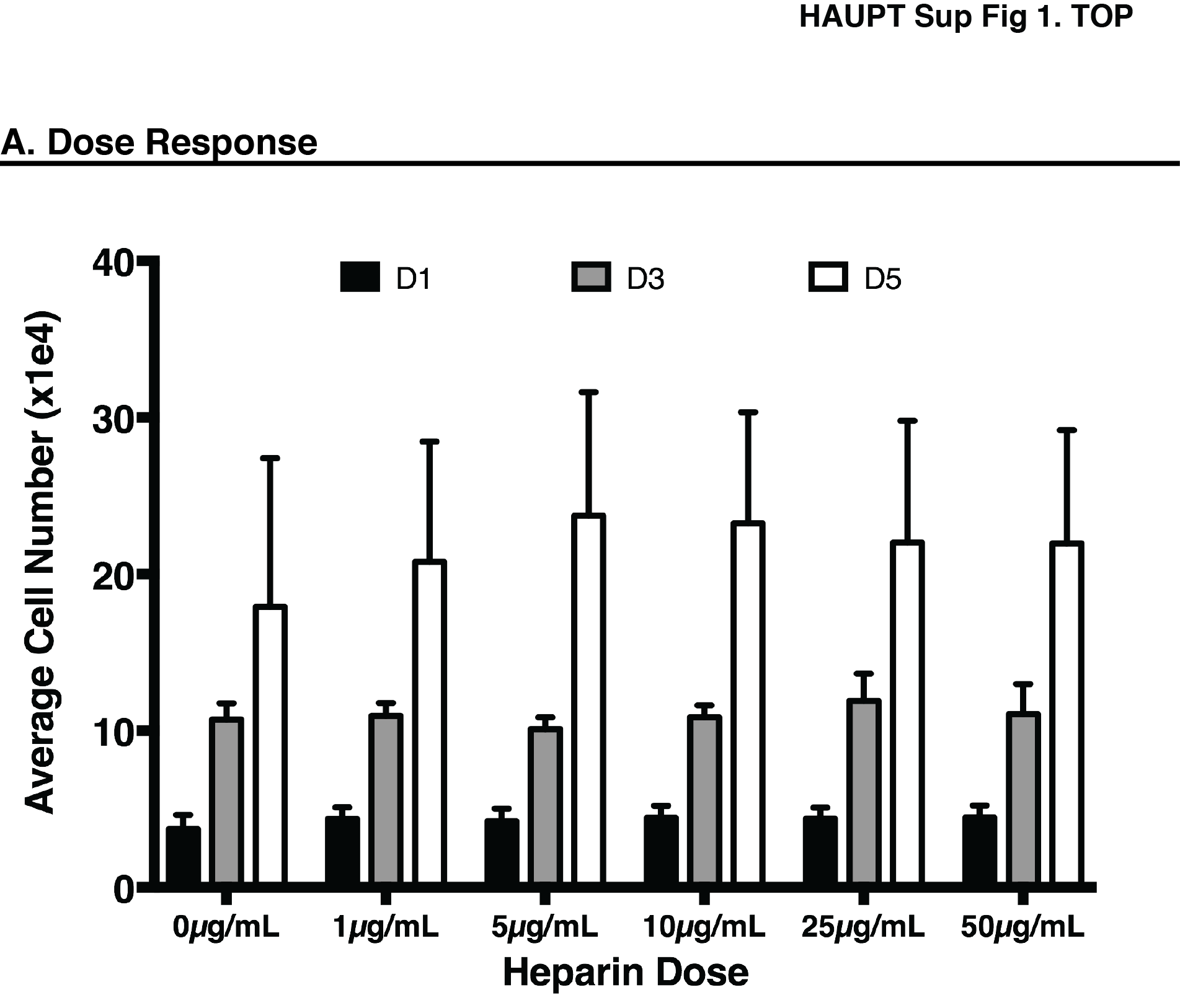


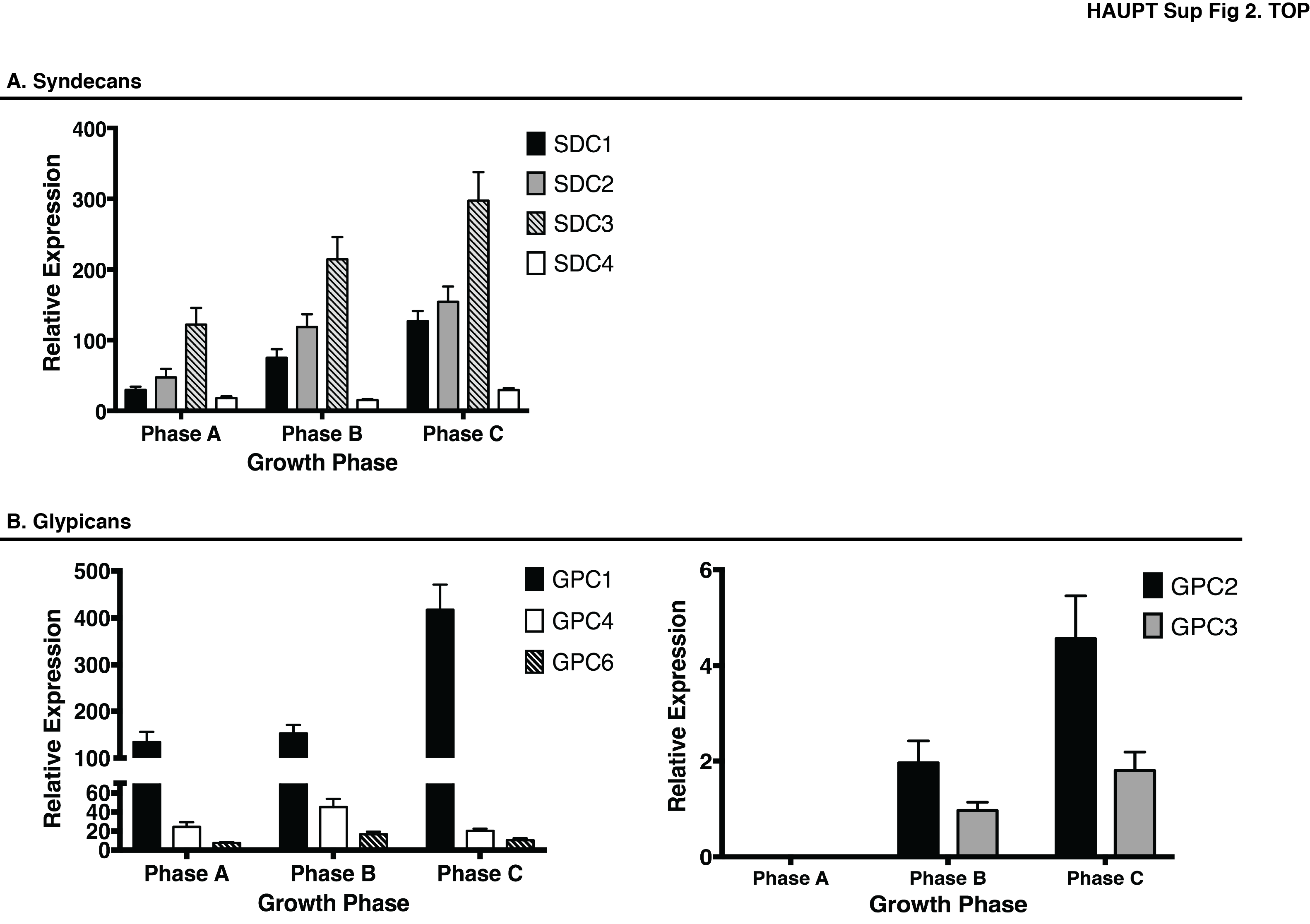


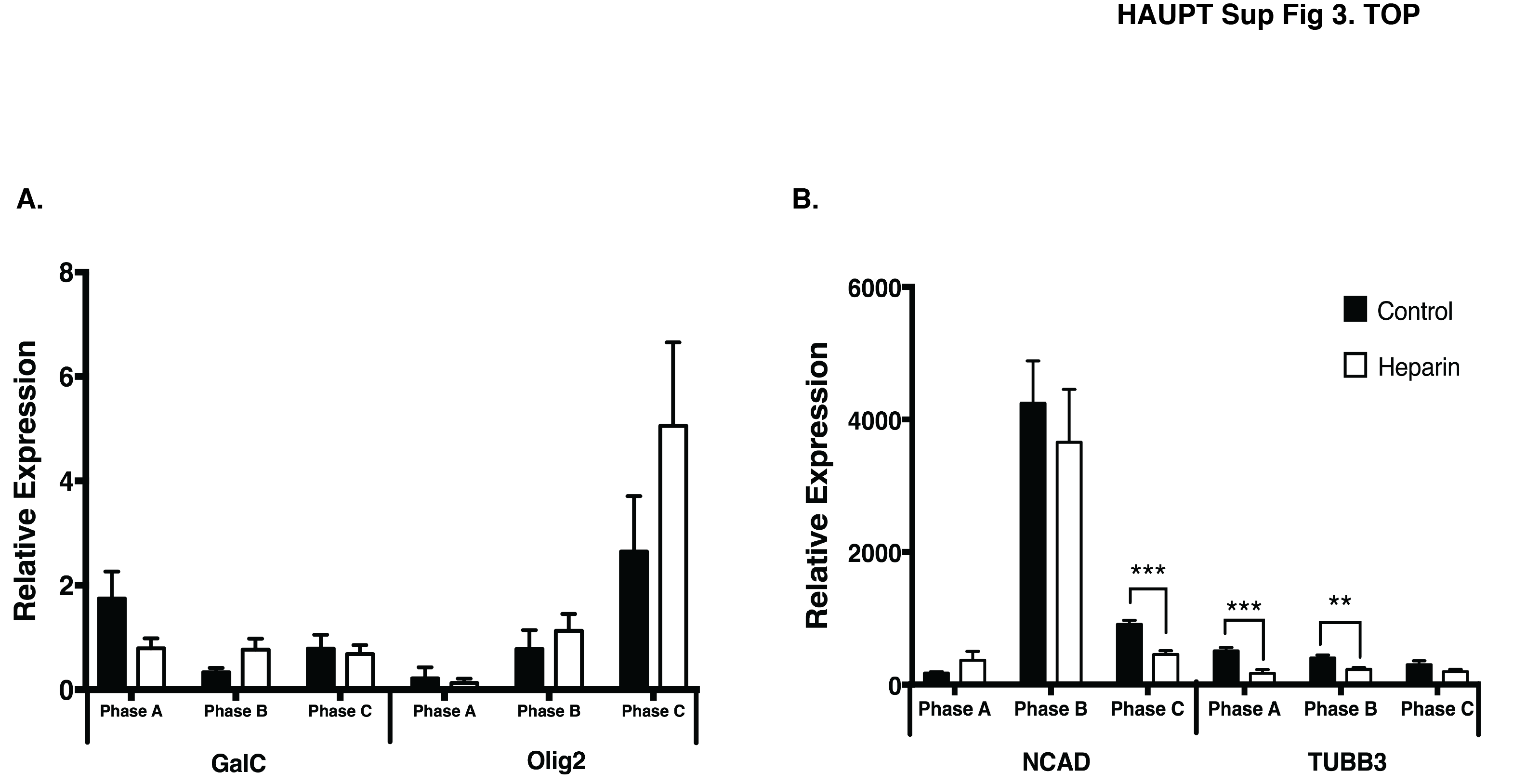


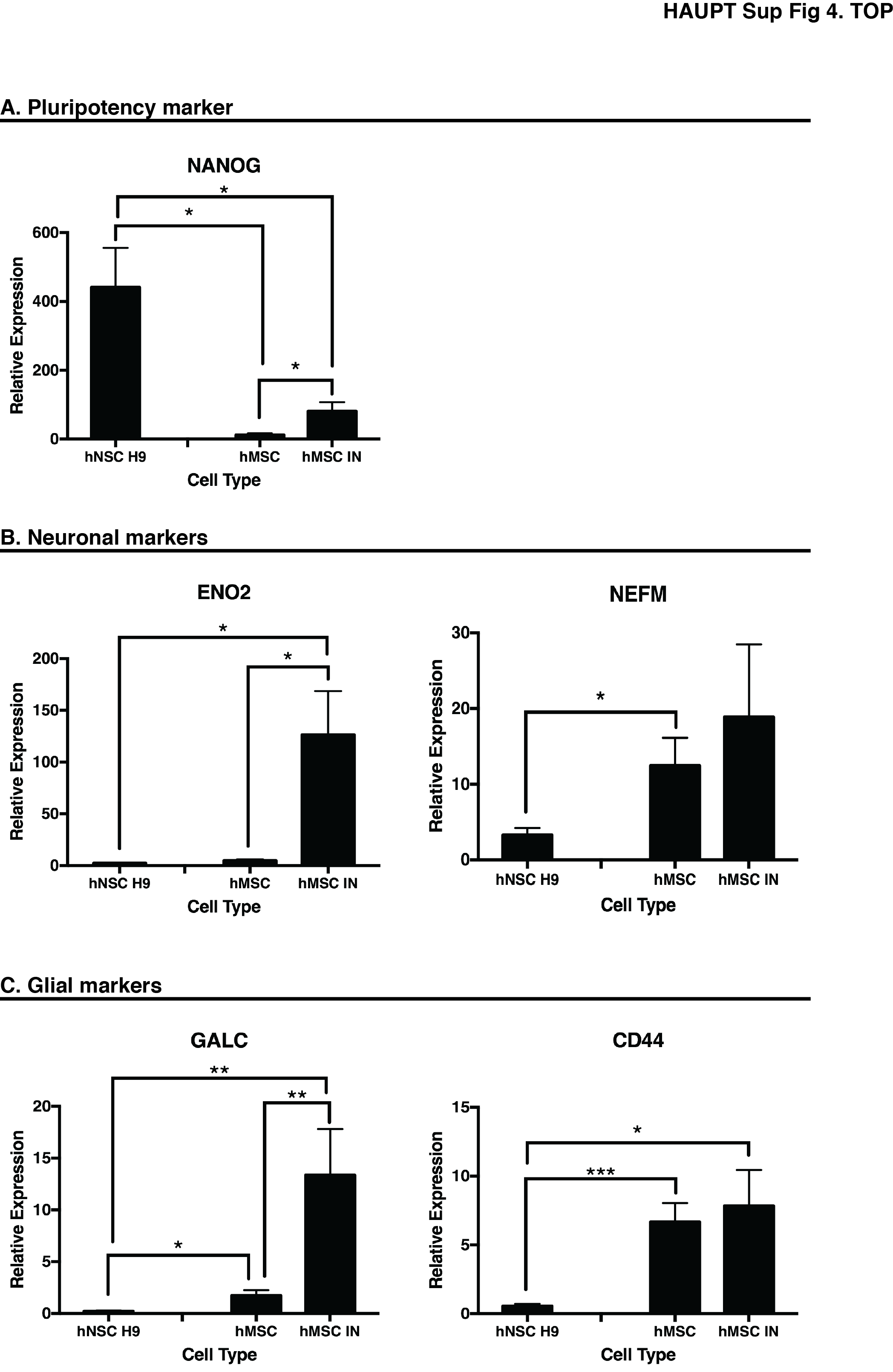


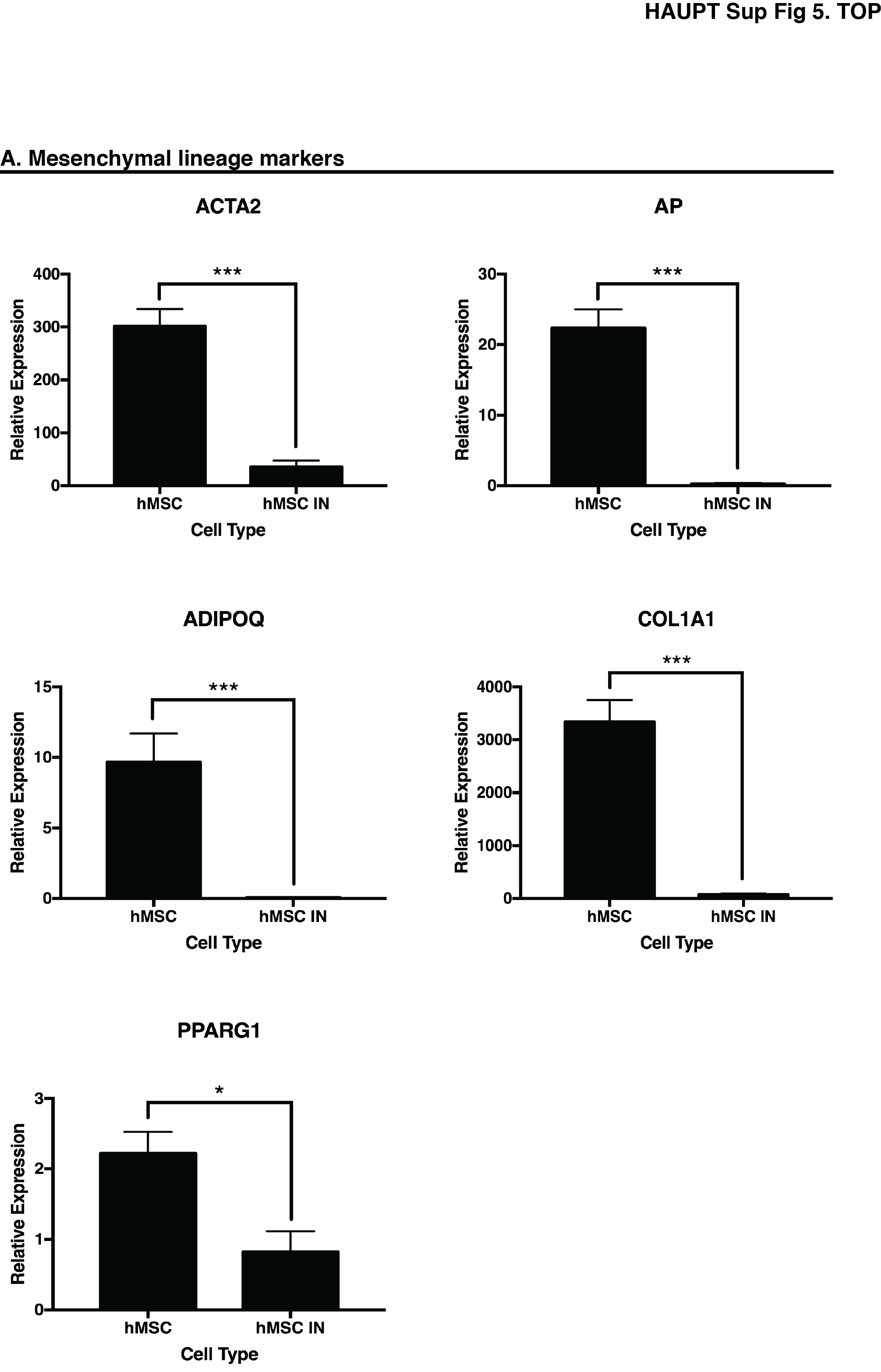

Supplement: TABLE S1 — Primer sequences used for Q-PCR analysis. [file Table_1.DOCX]
